# PARP7 inhibits type I interferon signaling to prevent autoimmunity and lung disease

**DOI:** 10.1084/jem.20241184

**Published:** 2025-02-19

**Authors:** Devon Jeltema, Kennady Knox, Nicole Dobbs, Zhen Tang, Cong Xing, Antonina Araskiewicz, Kun Yang, Ivan Rodriguez Siordia, Jason Matthews, Michael Cohen, Nan Yan

**Affiliations:** 1 Department of Immunology, University of Texas Southwestern Medical Center, Dallas, TX, USA; 2 Department of Chemical Physiology and Biochemistry, Oregon Health and Science University, Portland, OR, USA; 3 Department of Nutrition, Institute of Basic Medical Sciences, University of Oslo, Oslo, Norway

## Abstract

Type I IFN (IFN-I) induce hundreds of antiviral genes as well as negative regulators that limit IFN-I signaling. Here, we investigate the family of 16 PARPs and find that 11 PARPs are ISGs, of which 8 PARPs inhibit IFN-I production. PARP7 is the most potent negative feedback regulator of IFN-I production. Using *Parp7*^*−/−*^ and *Parp7*^*H532A/H532A*^ mice, we show that PARP7 loss leads to systemic autoimmunity characterized by splenomegaly and increased autoantibodies and inflammatory cytokines. PARP7 loss also results in perivascular immune infiltration in the lung that forms tertiary lymphoid structures. Mechanistically, PARP7 inhibits multiple innate immune pathways in a cell-intrinsic and MARylation-dependent manner. PARP7 interacts with IRF3 through the catalytic domain and disrupts the IRF3:CBP/p300 transcriptional holocomplex required for IFN-I production. *Irf3*^−/−^ or *Irf3*^*S1/S1*^ (transcription defective) or *Sting*^*−/−*^ rescues *Parp7*^*H532A/H532A*^ mouse autoimmunity and lung disease. Together, our study reveals physiological functions of PARP7 as a negative feedback regulator of IFN-I production that maintains immune homeostasis particularly in the lung.

## Teaser

Many PARPs are IFN-stimulated genes, of which PARP7 potently inhibits IFN signaling. Jeltema et al. show that *Parp7* deficiency in mice leads to systemic autoimmunity and lung disease that are mediated by IRF3 transcriptional activity.

## Graphical Abstract

**Figure:**
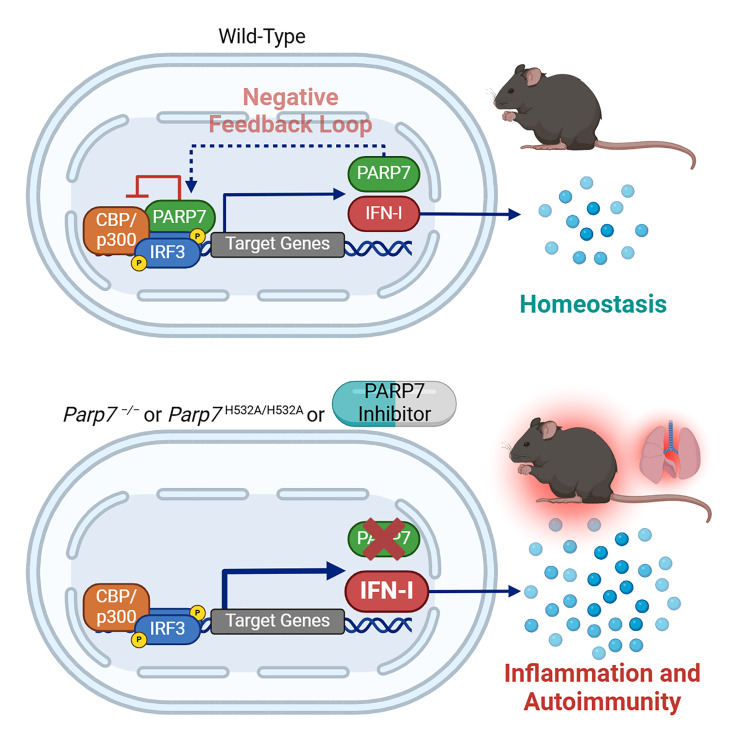

## Introduction

The innate immune system responds to invading pathogens and perturbations in cellular homeostasis by triggering the activation of type I IFN (IFN-I) and inflammatory responses. IFN-I is a powerful cytokine that plays a crucial role in protecting the host from pathogens and malignancies. Despite this protective function, uncontrolled production of IFN-I underlies the development of severe autoimmune and inflammatory diseases called type I interferonopathies (Crow and Stetson, 2022). This dilemma emphasizes the importance of maintaining a balance of IFN-I signaling to restrict infection without causing significant host damage. IFN-I induces the expression of hundreds of IFN-stimulated genes (ISGs), of which many function as antiviral effectors (Schoggins, 2019); however, a very small number of ISGs encode proteins that inhibit IFN-I signaling, thereby acting as a negative feedback loop. For example, USP18 and ISG15 are negative feedback regulators of IFNAR-JAK-STAT signaling, and their deficiency in humans drives severe autoimmune disease (Zhang et al., 2015; Meuwissen et al., 2016; Bogunovic et al., 2012; Martin-Fernandez et al., 2020; Malakhova et al., 2006). Whether additional negative feedback regulators of IFN-I signaling exist is unclear.

Poly-ADP-ribose polymerases (PARPs) are a family of 17 proteins (16 in mice) that catalyze the transfer of ADP-ribose from NAD^+^ to target substrates, which is a reversible posttranslational modification called ADP-ribosylation. PARPs are reported to function in various cellular stress responses, including DNA damage repair, stress granule formation, unfolded protein response, and viral infection (Bai, 2015). While PARP1/2 inhibitors are widely used as a cancer therapy in the clinic (Bhamidipati et al., 2023), focus is shifting to the development of inhibitors for other PARPs are therapies for cancer and other diseases (Kirby et al., 2018; Falchook et al., 2021; Schenkel et al., 2021; Eddie et al., 2022; Niepel et al., 2022; Yap et al., 2023; Wong et al., 2023). However, the physiological functions of many PARPs remain poorly characterized. This represents a critical gap in knowledge with potential for impact in patient care, especially when many PARP inhibitors are entering into clinical trials.

Here, we show that PARP7 is a negative feedback regulator of IFN-I production that inhibits the interaction of IFN regulatory factor 3 (IRF3) with transcriptional co-activators CREB-binding protein (CBP)/p300 in a mono-ADP-ribosylation (MARylation)-dependent manner, thereby resolving IRF3 transcriptional activity. Mice deficient in *Parp7* or carrying an enzymatic-dead mutant *Parp7* developed systemic autoimmunity and lung disease driven by IRF3 transcriptional activity. Our study reveals a previously unknown physiological function of PARP7 in maintaining immune homeostasis, particularly in the lung.

## Results

### PARP screen identifies PARP7 as an ISG and potent inhibitor of IFN-I production

**Figure 1.**
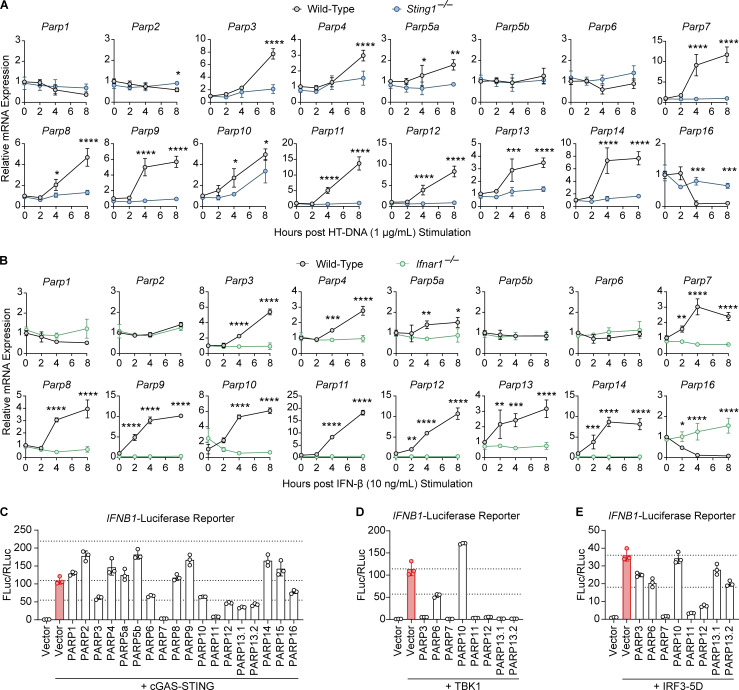
**PARP screen identifies PARP7 as an ISG and potent inhibitor of IFN-I production. (A)** qRT-PCR analysis of *Parp* gene expression in WT and *Sting1*^*−/−*^ BMDM following stimulation with HT-DNA (1 μg/ml; transfected) at indicated time points. **(B)** qRT-PCR analysis of *Parp* gene expression in WT and *Ifnar1*^*−/−*^ BMDM following stimulation with recombinant murine IFN-β (10 ng/ml) at indicated time points. **(C–E)** Quantification of luciferase activity in HEK293T cells transfected with *IFNB1* firefly luciferase, *TK*-Renilla luciferase, and other indicated plasmids for 24 h. Data are shown as mean ± SD. P values were determined by an ordinary two-way ANOVA (A and B). *P < 0.05, **P < 0.01, ***P < 0.001, and ****P < 0.0001. All data are representative of two independent experiments.

**Figure 2.**
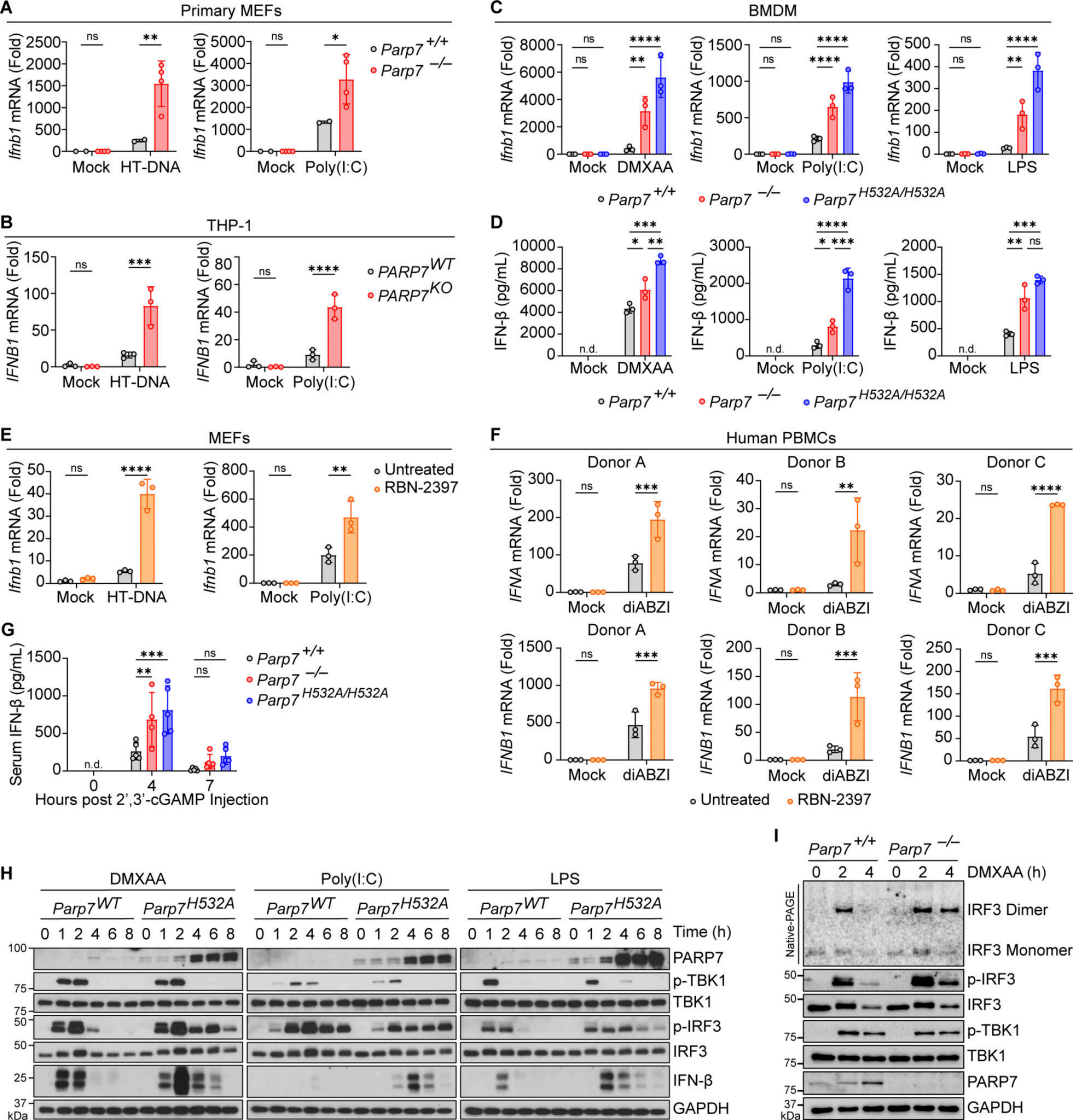
**PARP7 is a negative feedback ISG that inhibits IFN-I production. (A)** Primary MEFs from WT and *Parp7*^*−/−*^ littermates stimulated with HT-DNA (transfected; 1 μg/ml, 6 h) or Poly(I:C) (transfected; 1 μg/ml, 6 h). **(B)** qRT-PCR analysis of *IFNB1* expression in *PARP7*^*WT*^ and *PARP7*^*KO*^ THP-1 cells stimulated with HT-DNA (transfected; 1 μg/ml, 6 h) or Poly(I:C) (transfected; 1 μg/ml, 6 h). **(C)** qRT-PCR analysis of *Ifnb1* expression in WT, *Parp7*^*−/−*^, and *Parp7*^*H532A/H532A*^ BMDM stimulated with DMXAA (10 μg/ml, 4 h), Poly(I:C) (transfected; 1 μg/ml, 6 h), or LPS (100 ng/ml, 4 h). **(D)** ELISA analysis of mouse IFN-β in the supernatant of WT, *Parp7*^*−/−*^, and *Parp7*^*H532A/H532A*^ BMDM stimulated with DMXAA (10 μg/ml, 8 h), Poly(I:C) (transfected; 1 μg/ml, 8 h), or LPS (100 ng/ml, 8 h). **(E)** qRT-PCR analysis of *Ifnb1* in WT MEFs pre-treated with or without RBN-2397 (1 μM, 2 h) followed by stimulation with HT-DNA (transfected; 1 μg/ml, 6 h) or Poly(I:C) (transfected; 1 μg/ml, 6 h). **(F)** qRT-PCR analysis of *IFNA* and *IFNB1* expression in human PBMCs pre-treated with or without RBN-2397 (1 μM, 2 h) followed by stimulation with diABZI (500 nM, 4 h). **(G)** ELISA analysis of mouse IFN-β in the serum of in WT (*n* = 5), *Parp7*^*−/−*^ (*n* = 4), and *Parp7*^*H532A/H532A*^ (*n* = 5) mice at indicated time points after i.p. injection of 10 mg/kg 2′,3′-cGAMP. **(H)** Western blot time course analysis of signaling kinetics in WT and *Parp7*^*H532A/H532A*^ BMDM stimulated with DMXAA (10 μg/ml), Poly(I:C) (transfected; 1 μg/ml), or LPS (100 ng/ml). **(I)** Native-PAGE analysis of IRF3 dimer and western blot analysis of signaling events in WT and *Parp7*^*−/−*^ MEFs stimulated with DMXAA (10 μg/ml) at indicated time points. Data are shown as mean ± SD. P values were determined by two-way ANOVA (A–C and E–G) or one-way ANOVA (D). *P < 0.05, **P < 0.01, ***P < 0.001, ****P < 0.0001, and ns, not significant. All data are representative of at least three independent experiments. Source data are available for this figure: SourceData F2.

While several PARPs have reported functions in antiviral immunity (Zhu and Zheng, 2021), we performed a side-by-side comparison of the entire PARP family as potential regulators of IFN-I signaling. First, we examined the expression of all *Parp*s in two settings: (1) WT and stimulator of IFN genes (*Sting*)^*−/−*^ bone marrow–derived macrophage (BMDM) stimulated with an agonist HT-DNA that activates the cyclic GMP-AMP synthase (cGAS)–STING–IFN pathway (Fig. 1 A); (2) WT and *Ifnar*^*–/–*^ BMDM stimulated with recombinant IFN-β that activates the IFNAR–JAK–STAT pathway (Fig. 1 B). We found that the expression of *Parp3*, *Parp4*, *Parp5a*, *Parp7*, *Parp8*, *Parp9*, *Parp10*, *Parp11*, *Parp12*, *Parp13*, and *Parp14* was rapidly induced following stimulation with HT-DNA or rIFN-β, indicating that most (11 out of 16) PARPs are ISGs (Fig. 1, A and B). Interestingly, most IFN-inducible PARPs catalyze MARylation, including PARP3, PARP7, PARP8, PARP10, PARP11, PARP12, and PARP14. PARP4 catalyzes poly-ADP-ribosylation while PARP9 and PARP13 are inactive enzymes. PARP16 was the only IFN-repressed gene, whose only known function is in the unfolded protein response (Jwa and Chang, 2012). Collectively, these data demonstrate that most PARPs are ISGs, particularly those that catalyze MARylation, suggesting that PARPs play important roles in innate immunity.

Next, we used luciferase reporter assays to examine the ability of each PARP to regulate the production of IFN-β. In this assay, the transient expression of components of the cGAS–STING pathway activates the signaling cascade leading to the expression of an *IFNB1* promoter–driven firefly luciferase while an HSV thymidine kinase promoter–driven Renilla luciferase serves as a control. Activation of the *IFNB1* luciferase can be initiated from the top (e.g., cGAS-STING) or from downstream steps (e.g., TANK-binding kinase 1 [TBK1] and IRF3) to pinpoint which step of the signaling cascade is affected by a potential regulator. cGAS-STING expression led to robust activation of *IFNB1*-luciferase activity while the co-expression with PARP3, PARP6, PARP10, PARP12, PARP13.1, and PARP13.2 reduced the activity by 50%, suggesting these PARPs are moderate regulators of IFN-β production (Fig. 1 C). Remarkably, PARP7 and PARP11 strongly inhibited *IFNB1*-luciferase activity to <5% and 10%, respectively.

Next, we sought to determine which step of the innate immune signaling cascade these PARPs inhibited, we co-expressed TBK1 or IRF3-5D (a constitutively active IRF3 mutant) with these PARPs. We found that PARP3, PARP6, PARP7, PARP11, PARP12, and PARP13.2 retained their inhibition on both TBK1- and IRF3-5D–induced *IFNB1*-luciferase activity (Fig. 1, D and E). PARP13.1 retained its inhibition on TBK1-induced signaling but not IRF3-5D, while PARP10 lost its inhibition on both TBK1 and IRF3-5D. Overall, these data suggest that multiple PARPs are negative feedback regulators of IFN-β production that act at different steps of the signaling cascade, highlighting the importance of PARPs in maintaining immune homeostasis. We chose to further investigate PARP7 since it was the most potent negative feedback regulator of IFN-β production.

### PARP7 is a negative feedback ISG that inhibits IFN-I production

**Figure S1.**
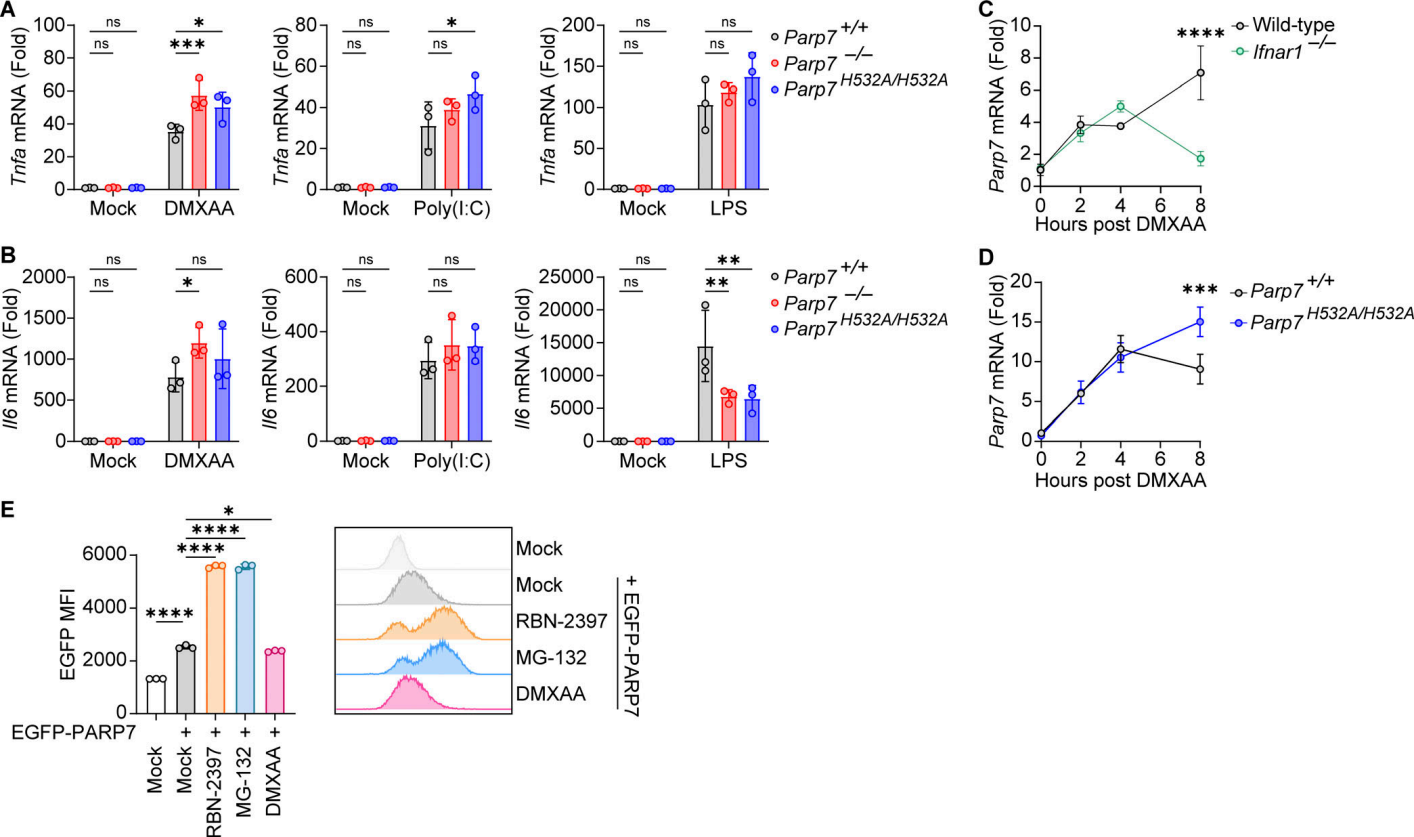
**PARP7 does not affect NF-κB signaling and analysis of PARP7 mRNA expression and protein stability. (A and B)** qRT-PCR analysis of *Tnfa* (A) or *Il6* (B) expression in WT, *Parp7*^*−/−*^, and *Parp7*^*H532A/H532A*^ BMDM stimulated with DMXAA (10 μg/ml, 4 h), Poly(I:C) (transfected; 1 μg/ml, 6 h), or LPS (100 ng/ml, 4 h). **(C)** qRT-PCR analysis of *Parp7* expression in WT and *Ifnar1*^*−/−*^ BMDM stimulated with DMXAA (10 μg/ml) for indicated time points. **(D)** qRT-PCR analysis of *Parp7* expression in WT and *Parp7*^*H532A/H532A*^ BMDM stimulated with DMXAA (10 μg/ml) for indicated time points. **(E)** Flow cytometric analysis of EGFP in *Parp7*^*−/−*^ MEFs reconstituted with CMV promoter–driven EGFP-PARP7 that were treated with RBN-2397 (1 μM, 24 h), MG-132 (10 μM, 6 h), or DMXAA (10 μg/ml, 6 h). Data are shown as mean ± SD. P values were determined by two-way ANOVA (A–D) or one-way ANOVA (E). *P < 0.05, **P < 0.01, ***P < 0.001, ****P < 0.0001, and ns, not significant. Data are representative of three independent experiments.

To confirm the findings of our screen, we measured *Ifnb1* expression in WT and *Parp7*^*−/−*^ primary MEFs transfected with HT-DNA (a cGAS-STING agonist) or Poly(I:C) (a retinoic acid–inducible gene I/melanoma differentiation-associated protein 5 agonist). The expression of *Ifnb1* was increased in *Parp7*^*−/−*^ MEFs compared with WT littermate control MEFs (Fig. 2 A). Similarly, *IFNB1* expression was increased in *PARP7*^*KO*^ THP-1 cells (human monocytes) transfected with HT-DNA or Poly(I:C) (Fig. 2 B). To determine whether this phenotype was dependent on PARP7 MARylation activity, we measured *Ifnb1* expression and IFN-β protein in BMDM isolated from WT, *Parp7*^*−/−*^, and *Parp7*^*H532A/H532A*^ (enzymatic-dead mutant) mice stimulated with DMXAA (a mouse STING agonist), transfected Poly(I:C), or LPS (a TLR4 agonist). Both *Ifnb1* mRNA expression and IFN-β protein were increased in *Parp7*^*−/−*^ and *Parp7*^*H532A/H532A*^ BMDM stimulated with either agonist (Fig. 2, C and D). In contrast, NF-κB target genes (e.g., *Tnfa* and *Il6*) did not consistently change in *Parp7*^*−/−*^ and *Parp7*^*H532A/H532A*^ cells compared with WT (Fig. S1, A and B). Many ISGs that are inducible by IFN-IFNAR can also be induced cell intrinsically after innate immune activation. We stimulated WT and *Ifnar1*^*−/−*^ BMDMs with STING agonist DMXAA and found that *Parp7* expression was induced in both cells, although *Parp7* induction in *Ifnar1*^*−/−*^ BMDMs peaked earlier and reached lower levels compared with WT BMDMs (Fig. S1 C).

Additionally, we treated WT cells with RBN-2397, which is a selective small molecule inhibitor of PARP7 that binds to the NAD^+^-binding pocket, inhibiting its MARylation activity (Gozgit et al., 2021). MEFs pre-treated with RBN-2397 had increased *Ifnb1* expression following stimulation with HT-DNA or Poly(I:C) (Fig. 2 E). We also isolated human peripheral blood mononuclear cells (PBMCs) from three healthy donors, pre-treated with or without RBN-2397 for 2 h, then stimulated with mock or diABZI. RBN-2397 pre-treatment increased diABZI-induced *IFNA* and *IFNB1* expression (Fig. 2 F). Next, we examined whether PARP7 inhibited IFN-β production in vivo. We injected WT, *Parp7*^*−/−*^, and *Parp7*^*H532A/H532A*^ mice with the endogenous STING agonist 2′,3′-cGAMP and measured levels of serum IFN-β 4 h later. Both *Parp7*^*−/−*^ and *Parp7*^*H532A/H532A*^ mice had increased levels of serum IFN-β compared with WT mice (Fig. 2 G). Together, these data suggest that PARP7 inhibits IFN-β production downstream of multiple innate sensing pathways in a MARylation-dependent manner.

We next analyzed the kinetics of TBK1–IRF3–IFN signaling events in BMDM stimulated with DMXAA, transfected Poly(I:C), and LPS. We found that WT and *Parp7*^*H532A/H532A*^ BMDM had comparable levels of p-TBK1 after stimulation; however, *Parp7*^*H532A/H532A*^ BMDM displayed increased p-IRF3 levels at later time points compared with WT, which corresponds with increased levels of IFN-β protein in *Parp7*^*H532A/H532A*^ BMDM (Fig. 2 H). Similarly, increased p-IRF3 and IRF3 dimer was observed in *Parp7*^*−/−*^ MEFs (Fig. 2 I). Further, the sustained levels of p-IRF3, despite no difference in p-TBK1 levels in PARP7-deficient cells, suggest that PARP7 functions in the resolution, rather than activation, of p-IRF3 leading to increased IFN-I production.

Interestingly, PARP7 protein levels were higher in *Parp7*^*H532A/H532A*^ cells compared with WT cells, especially after innate immune agonist stimulation. *Parp7* mRNA levels were similar at 2 and 4 h after DMXAA stimulation, but higher at 8 h after stimulation (Fig. S1 D). Previous studies showed that the inhibition of PARP7 enzymatic activity increases its stability (Kamata et al., 2021; Sanderson et al., 2022). To test this, we reconstituted *Parp7*^*−/−*^ MEFs with CMV promoter–driven EGFP-PARP7 and treated cells with RBN-2397, MG-132, or DMXAA. Treatment with RBN-2397 or MG-132 increased EGFP-PARP7 protein levels as measured by EGFP, confirming that PARP7 is degraded by the proteasome, which requires its enzymatic activity. Stimulation with DMXAA did not alter EGFP-PARP7 protein level (Fig. S1 E). These data suggest that the higher PARP7-H532A protein level in *Parp7*^*H532A/H532A*^ BMDMs compared with endogenous PARP protein level in WT BMDMs after innate agonist stimulation is due to increased mutant protein stability and increased *Parp7-H532A* mRNA expression.

### PARP7 loss-of-function leads to tonic IFN-I signaling

**Figure 3.**
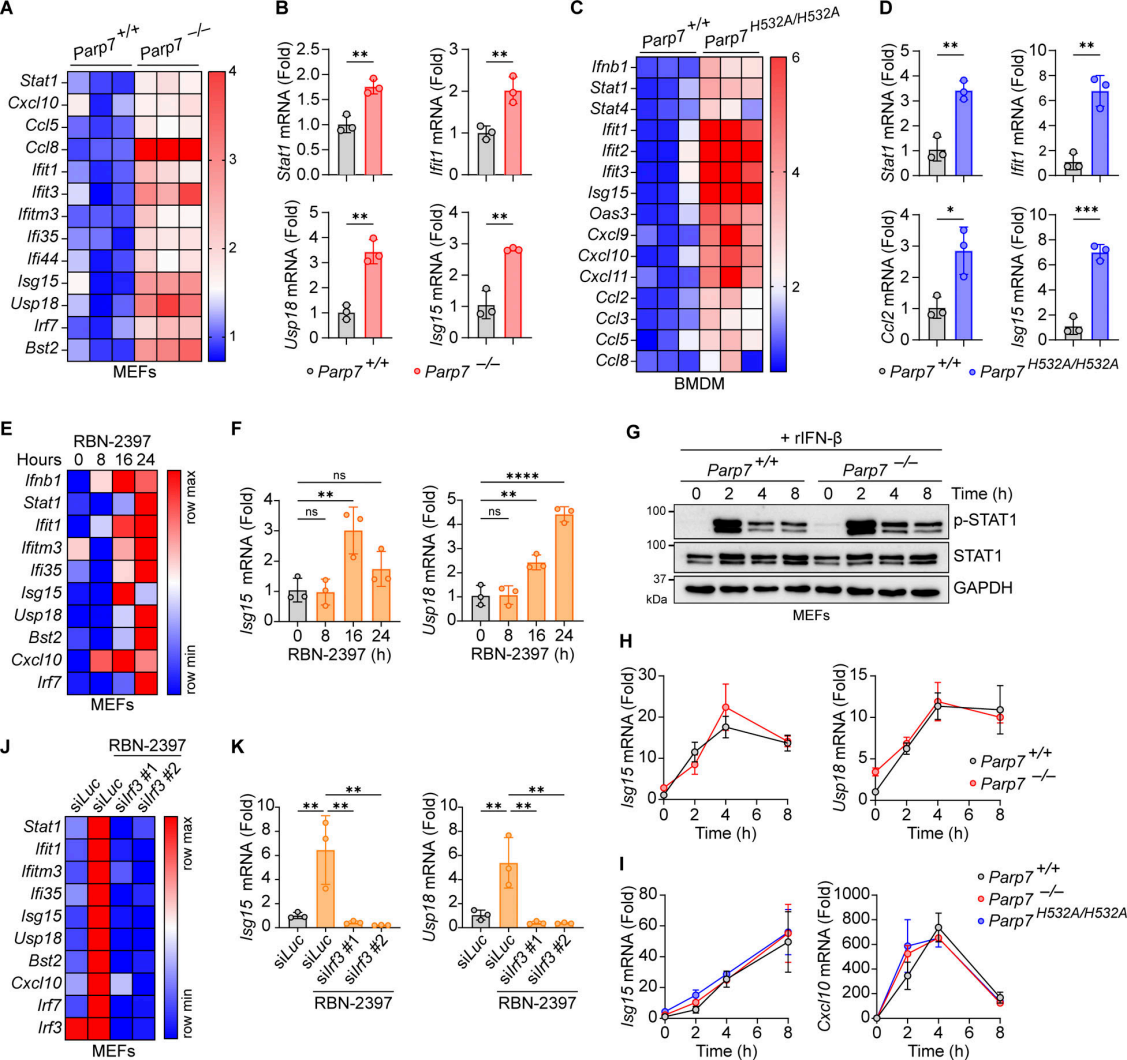
**PARP7 loss-of-function increases tonic IFN-I signaling. (A)** A heat map showing the tonic expression of various ISGs in WT and *Parp7*^*−/−*^ MEFs. **(B)** Representative bar graphs of individual ISGs from A. **(C)** A heat map showing the tonic expression of various ISGs in WT and *Parp7*^*H532A/H532A*^ BMDM. **(D)** Representative bar graphs of individual ISGs from C. **(E)** A heat map showing the expression of various ISGs in MEFs treated with RBN-2397 (1 μM) for indicated time points. **(F)** Representative bar graphs of individual ISGs from E. **(G)** Western blot analysis of p-STAT1 and STAT1 in WT and *Parp7*^*−/−*^ MEFs stimulated with recombinant murine IFN-β (10 ng/ml) for indicated time points. **(H)** Expression of *Isg15* and *Usp18* in WT and *Parp7*^*−/−*^ MEFs stimulated with recombinant murine IFN-β (10 ng/ml) for indicated time points. **(I)** Expression of *Isg15* and *Cxcl10* in WT and *Parp7*^*H532A/H532A*^ BMDM stimulated with recombinant murine IFN-β (10 ng/ml) for indicated time points. **(J)** A heat map showing the expression of various ISGs in MEFs treated with RBN-2397 (1 μM, 24 h) following siRNA knockdown of *Irf3* or *Luc* (control). **(K)** Representative bar graphs of individual ISGs from J. Data are shown as mean ± SD. P values were determined by Student’s *t* test (B and D), one-way ANOVA (F and K), or two-way ANOVA (H and I). *P < 0.05, **P < 0.01, ***P < 0.001, ****P < 0.0001, and ns, not significant. All data are representative of three independent experiments. Source data are available for this figure: SourceData F3.

Next, we examined the effect of PARP7 deficiency on tonic IFN-I signaling by measuring the resting state expression of various ISGs. Both *Parp7*^*−/−*^ and *Parp7*^*H532A/H532A*^ cells had increased expression of various ISGs compared with WT cells, suggesting that PARP7 loss-of-function increases tonic IFN-I signaling (Fig. 3, A–D). Similarly, MEFs treated with RBN-2397 had increased tonic ISG expression at 16 and 24 h (Fig. 3, E and F). Together these data suggest that PARP7 is important in suppressing homeostatic IFN-I signaling.

Since ISGs can be induced cell intrinsically by IRF3 or cell extrinsically by JAK-STAT following IFN-I binding to autocrine or paracrine receptors, we assessed whether PARP7 directly inhibits the JAK–STAT pathway. There was no difference in p-STAT1 or STAT1 levels between WT and *Parp7*^*−/−*^ MEFs following stimulation with recombinant IFN-β (Fig. 3 G). Additionally, we observed no difference in the expression of various ISGs between WT, *Parp7*^*−/−*^, and *Parp7*^*H532A/H532A*^ cells stimulated with recombinant IFN-β (Fig. 3, H and I). Since PARP7 does not directly affect JAK-STAT signaling, we tested whether the increased tonic ISG expression in RBN-2397 treated cells was dependent on upstream IFN-I production by knocking down IRF3. The knockdown of IRF3 ablated the increased tonic ISG expression induced by RBN-2397 treatment (Fig. 3, J and K). Collectively, these data suggest that PARP7 loss-of-function increases tonic IFN-I signaling in an IRF3-dependent manner.

### PARP7 loss-of-function in mice induces systemic autoimmunity

**Figure S2.**
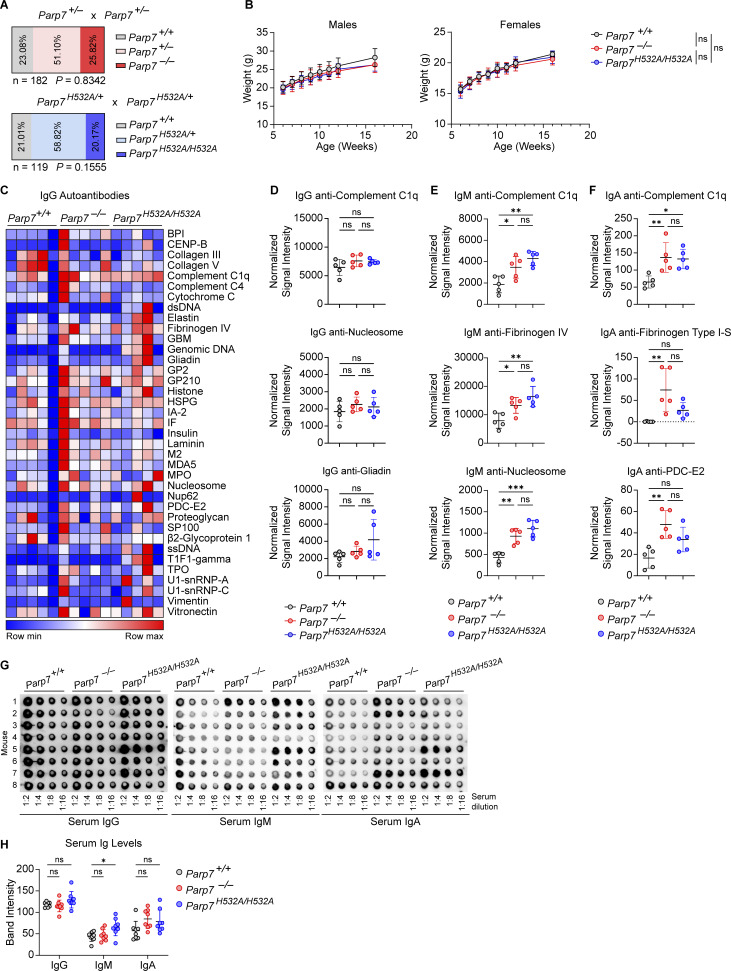
**Mendelian ratios, body weights, and autoantibodies in WT, *Parp7*** ^***−/−***^ **, and *Parp7*** ^***H532A/H532A***^ **mice. (A)** Observed Mendelian ratios from crosses of *Parp7*^*−/+*^ or *Parp7*^*H532A/+*^ mice. **(B)** Body weight of *Parp7*^+/+^ (*n* = 10 male, 10 female), *Parp7*^*−/−*^ (*n* = 10 male, 9 female), and *Parp7*^*H532A/H532A*^ (*n* = 8 male, 8 female) mice. **(C)** A heat map showing IgG autoantibody array analysis of mouse serum from 10-mo-old WT, *Parp7*^*−/−*^, and *Parp7*^*H532A/H532A*^ mice (*n* = 5). **(D)** Representative bar graphs of individual IgG autoantibodies from C. **(E)** Representative bar graphs of individual IgM autoantibodies from Fig. 4 D. **(F)** Representative bar graphs of individual IgA autoantibodies from Fig. 4 E. **(G)** A dot blot analysis of total IgG, IgM, and IgA in mouse serum from 10-mo-old WT, *Parp7*^*−/−*^, and *Parp7*^*H532A/H532A*^ mice (*n* = 8). **(H)** Quantification of serum Ig levels from G. Data are shown as mean ± SD. P values were determined by Chi-square test (A), two-way ANOVA (B), or one-way ANOVA (D–F and H). *P < 0.05, **P < 0.01, ***P < 0.001, ****P < 0.0001, and ns, not significant. Data are pooled from at least two independent experiments.

**Figure 4.**
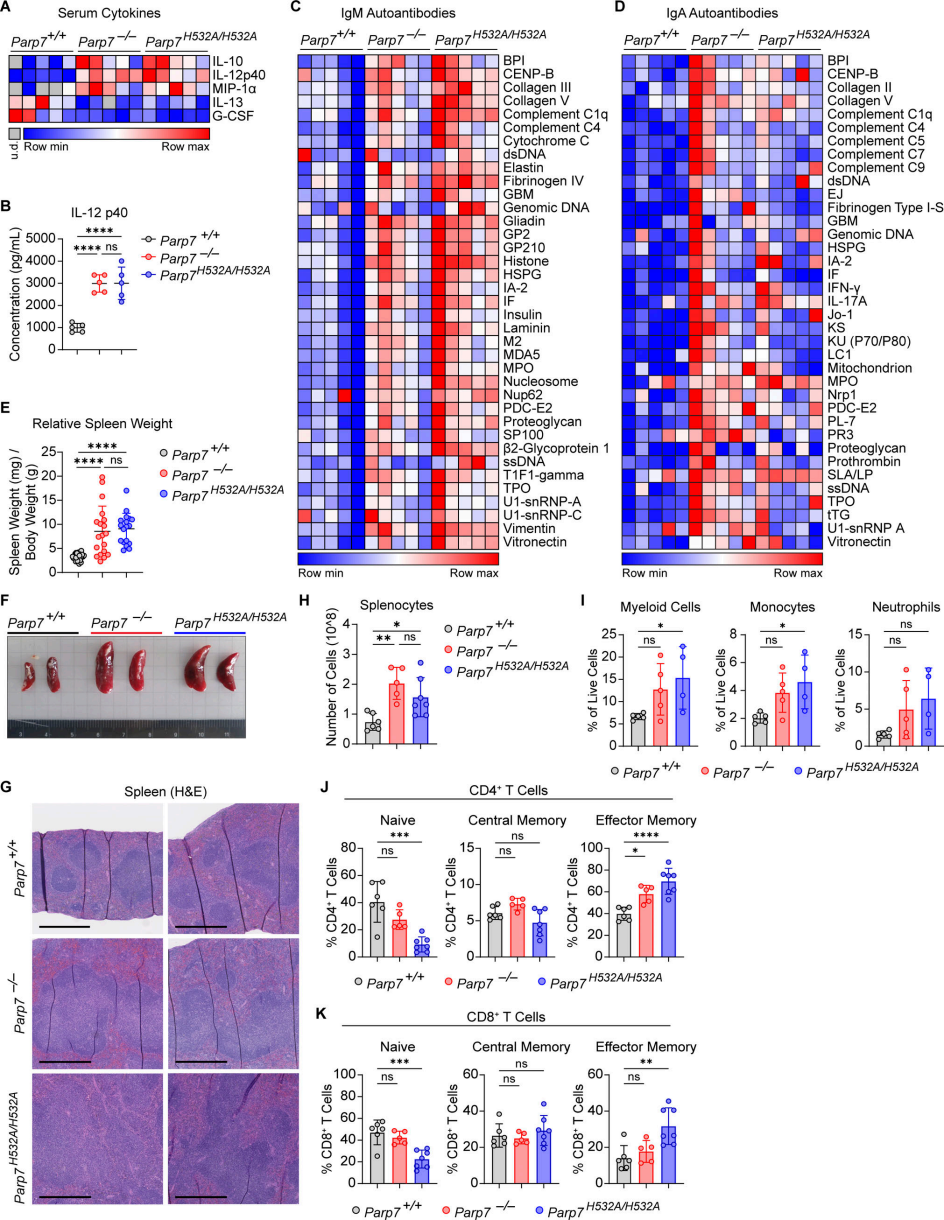
**PARP7 loss-of-function mice develop serological autoimmunity. (A)** A heat map of the serum cytokines in 10-mo-old WT, *Parp7*^*−/−*^, and *Parp7*^*H532A/H532A*^ mice (*n* = 5). **(B)** Graph of serum IL-12 p40 in 10-mo-old WT, *Parp7*^*−/−*^, and *Parp7*^*H532A/H532A*^ mice (*n* = 5). **(C)** A heat map showing IgM autoantibody array analysis of mouse serum from 10-mo-old WT, *Parp7*^*−/−*^, and *Parp7*^*H532A/H532A*^ mice (*n* = 5). **(D)** A heat map showing IgA autoantibody array analysis of mouse serum from 10-mo-old WT, *Parp7*^*−/−*^, and *Parp7*^*H532A/H532A*^ mice (*n* = 5). **(E)** Bar graph showing the relative spleen weight of 10-mo-old WT (*n* = 29), *Parp7*^*−/−*^ (*n* = 19), and *Parp7*^*H532A/H532A*^ (*n* = 18) mice. **(F)** Representative image of spleens from 10-mo-old WT, *Parp7*^*−/−*^, and *Parp7*^*H532A/H532A*^ mice. **(G)** Representative H&E staining of the spleen from 10-mo-old WT, *Parp7*^*−/−*^, and *Parp7*^*H532A/H532A*^ mice. Bar, 500 μM. **(H)** Bar graph showing the total number of splenocytes from 10-mo-old WT (*n* = 6), *Parp7*^*−/−*^ (*n* = 5), and *Parp7*^*H532A/H532A*^ (*n* = 7) mice. **(I)** Bar graphs showing the percentage of splenic myeloid cells, monocytes, and neutrophils 8-mo-old WT (*n* = 5), *Parp7*^*−/−*^ (*n* = 5), and *Parp7*^*H532A/H532A*^ (*n* = 4) mice. **(J and K)** Bar graphs showing the percentage of splenic CD4^+^ (J) or CD8^+^ (K) T cell populations in 10-mo-old WT (*n* = 6), *Parp7*^*−/−*^ (*n* = 5), and *Parp7*^*H532A/H532A*^ (*n* = 7) mice. Data are shown as mean ± SD. P values were determined by one-way ANOVA (B, E, and H–K). *P < 0.05, **P < 0.01, ***P < 0.001, ****P < 0.0001, and ns, not significant. Data are representative of at least two independent experiments. Flow cytometry data are pooled from two independent cohorts of mice.

Next, we investigated the physiological function of PARP7 in vivo by comparing WT, *Parp7*^*−/−*^, and *Parp7*^*H532A/H532A*^ mice. All three genotypes were born at the expected Mendelian ratio and have similar body weights (Fig. S2, A and B). We measured serum cytokines and autoantibodies at 10 mo old to assess for serological autoimmunity. Both *Parp7*^*−/−*^ and *Parp7*^*H532A/H532A*^ mice had elevated serum cytokines such as IL-12 p40 (Fig. 4, A and B). Additionally, both *Parp7*^*−/−*^ and *Parp7*^*H532A/H532A*^ mice had significantly higher levels of IgM and IgA, but not IgG, autoantibodies against a wide range of antigens commonly associated with lupus and other autoimmune diseases (Fig. 4, C and D; and Fig. S2, C–H). The elevated production of autoantibodies is likely a reflection of polyclonal B cell stimulation.

**Figure S3.**
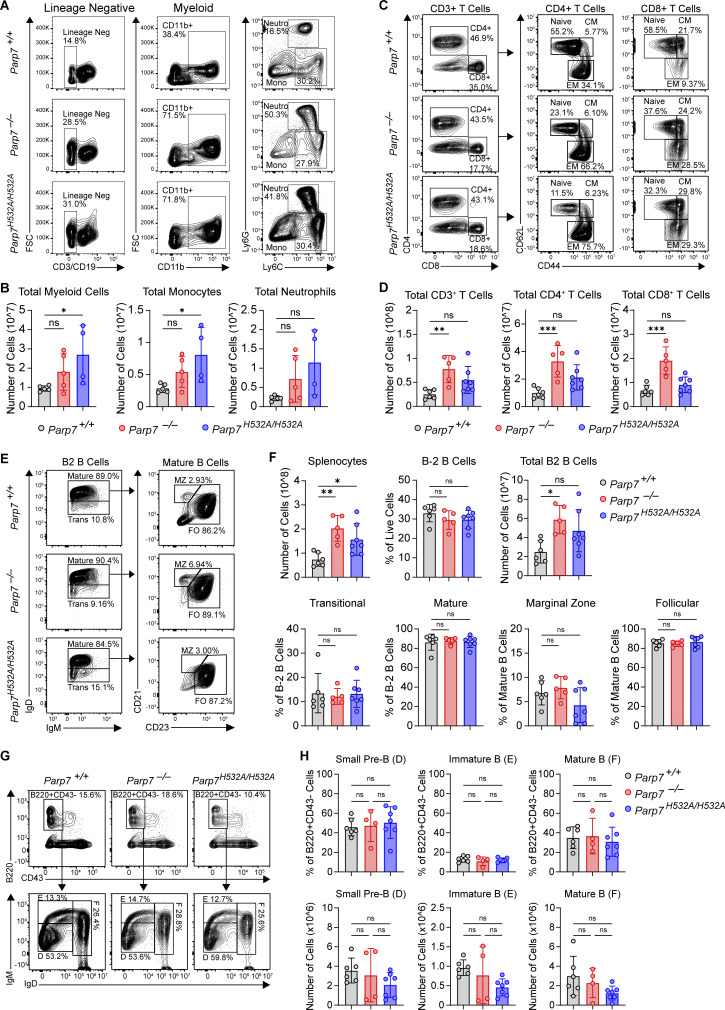
**Flow cytometry analysis of B cell and myeloid populations. (A)** Representative flow plots of splenic T cell populations from Fig. 4 J. **(B)** Bar graphs of total myeloid cells, monocytes, and neutrophils from the spleen of 8-mo-old WT, *Parp7*^*−/−*^, and *Parp7*^*H532A/H532A*^ mice. **(C)** Representative flow plots from Fig. 4, K and L. **(D)** Bar graphs of total T cells, CD4^+^ T cells, and CD8^+^ T cells in the spleen of 10-mo-old WT, *Parp7*^*−/−*^, and *Parp7*^*H532A/H532A*^ mice. **(E)** Representative flow plots of splenic B cell populations from Fig. S3 F. **(F)** Bar graph showing the percentage of splenic B cell populations and total B2-B cells in the spleen of 10-mo-old WT, *Parp7*^*−/−*^, and *Parp7*^*H532A/H532A*^ mice. **(G)** Representative flow plots of bone marrow B cell populations from Fig. S3 H. **(H)** Bar graph showing the percentage of bone marrow B cell populations in 10-mo-old WT, *Parp7*^*−/−*^, and *Parp7*^*H532A/H532A*^ mice. Data are shown as mean ± SD. P values were determined by one-way ANOVA (B, D, F, and H). *P < 0.05, **P < 0.01, ***P < 0.001, ****P < 0.0001, and ns, not significant. Flow cytometry data are pooled from at least two independent cohorts of mice.

Additionally, we found that *Parp7*^*−/−*^ and *Parp7*^*H532A/H532A*^ mice developed splenomegaly with a 2.5-fold increase in spleen weights, an expansion of white pulp, and disrupted splenic architecture (Fig. 4, E–H). To determine the cell population(s) responsible for the splenomegaly, we performed flow cytometry analysis on various immune cell populations in the spleen. First, we found an expansion in the myeloid compartment with increased percentages and total numbers of monocytes (Fig. 4 I and Fig. S3, A and B). *Parp7*^*−/−*^ and *Parp7*^*H532A/H532A*^ mice also had increased effector memory and reduced naïve T cells consistent with systemic autoimmunity and chronic inflammation (Fig. 4, J and K; and Fig. S3, C and D). Lastly, there were no differences in the percentages of various B cell populations in the spleen; however, the total number of B2 B cells in the spleen were increased (Fig. S3, E and F). We observed no differences in late B cell development in the bone marrow either (Fig. S3, G and H). Together, these data demonstrate that *Parp7*^*−/−*^ and *Parp7*^*H532A/H532A*^ mice develop systemic autoimmunity.

### PARP7 maintains immune homeostasis in the lung

**Figure S4.**
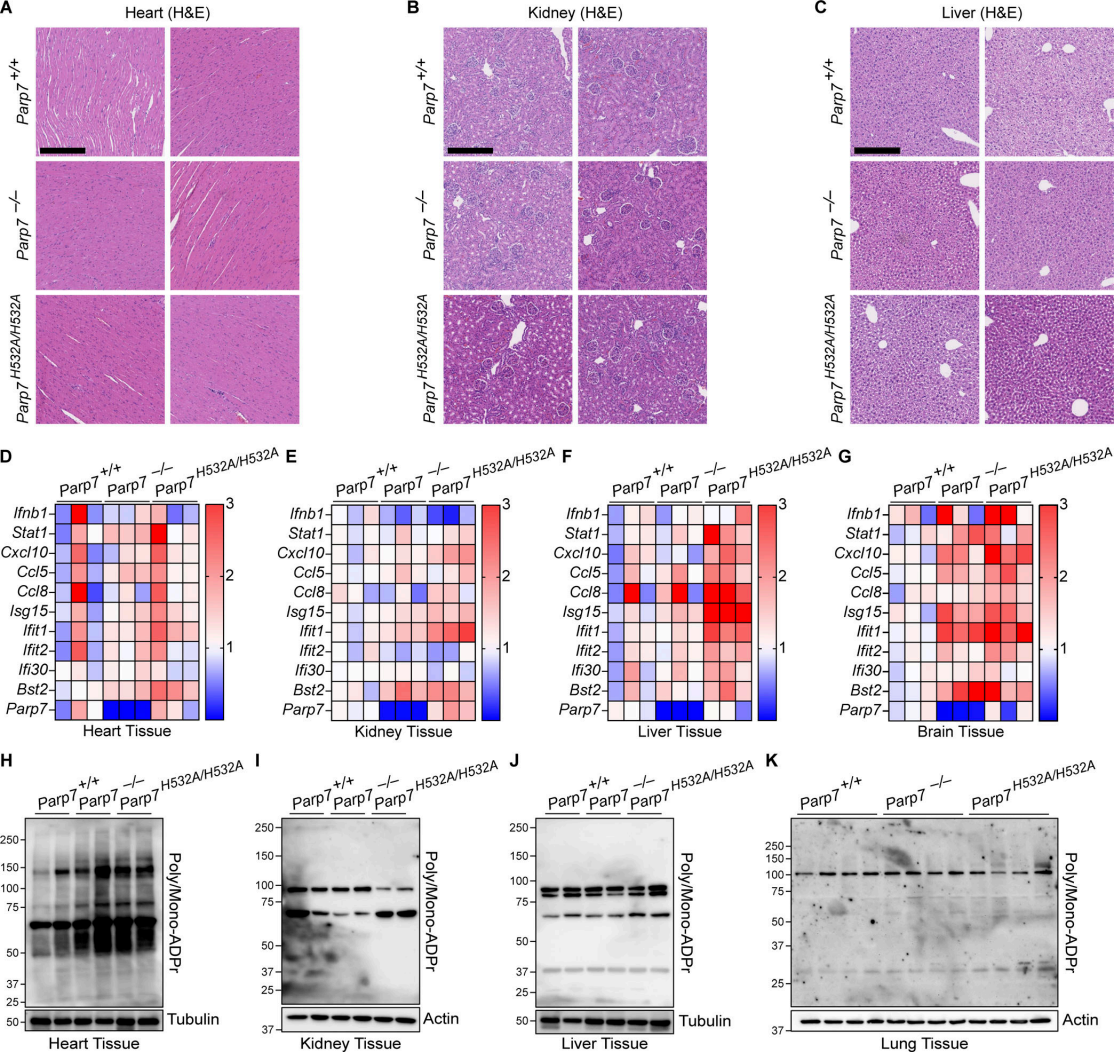
**Tissue pathology, ISG signature, and ADP-ribose levels. (A–C)** Representative H&E staining of the heart (A), kidney (B), and liver (C) from 10-mo-old WT, *Parp7*^*−/−*^, and *Parp7*^*H532A/H532A*^ mice. **(D–G)** A heat map showing qRT-PCR analysis of the expression of various ISGs (left) in the heart (D), kidney (E), liver (F), and brain (G) from 10-mo-old WT, *Parp7*^*−/−*^, and *Parp7*^*H532A/H532A*^ mice (*n* = 3). **(H–K)** Western blot analysis of whole tissue ADP-ribose levels in the heart (H), kidney (I), liver (J), and lung (K) from WT, *Parp7*^*−/−*^, and *Parp7*^*H532A/H532A*^ mice (*n* = 3 for heart, kidney, and liver or *n* = 4 for lung). Data are representative of two independent experiments. Source data are available for this figure: SourceData FS4.

**Figure 5.**
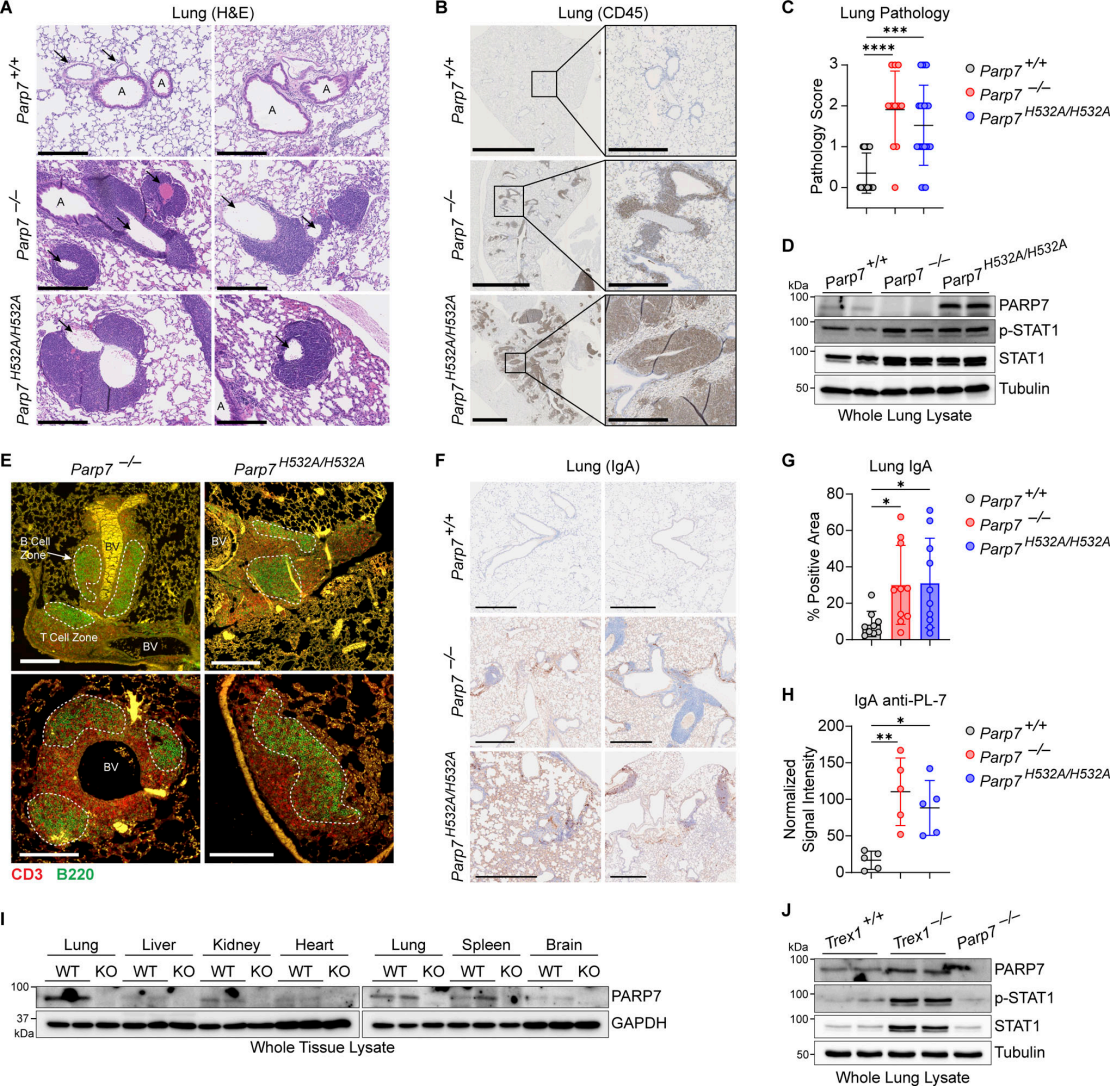
**PARP7 maintains lung immune homeostasis. (A)** Representative H&E staining of the lung from 10-mo-old WT, *Parp7*^*−/−*^, and *Parp7*^*H532A/H532A*^ mice. Black arrows, blood vessels; A, airways. Scale bars, 500 μm. **(B)** Representative IHC staining of CD45 in the lung from 10-mo-old WT, *Parp7*^*−/−*^, and *Parp7*^*H532A/H532A*^ mice. Scale bars, 2.5 mm or 500 μm (inset). **(C)** Histopathological scoring of the lung from 10-mo-old WT (*n* = 17), *Parp7*^*−/−*^ (*n* = 11), and *Parp7*^*H532A/H532A*^ (*n* = 21) mice. **(D)** Western blot analysis of indicated protein from whole lung lysate from 10-mo-old WT, *Parp7*^*−/−*^, and *Parp7*^*H532A/H532A*^ mice. **(E)** Representative IF staining of T cells (CD3, red) and B cells (B220, green) in the lung from 10-mo-old WT, *Parp7*^*−/−*^, and *Parp7*^*H532A/H532A*^ mice. B cell zones are outlined by white dashed line. BV, blood vessels. Scale bars, 200 μm. **(F)** Representative IHC staining of IgA in the lung from 10-mo-old WT, *Parp7*^*−/−*^, and *Parp7*^*H532A/H532A*^ mice. Scale bars, 500 μm. **(G)** Percent IgA positive area of the lung from 10-mo-old WT (*n* = 9), *Parp7*^*−/−*^ (*n* = 10), and *Parp7*^*H532A/H532A*^ (*n* = 10) mice. **(H)** Serum IgA anti-PL-7 from 10-mo-old WT, *Parp7*^*−/−*^, and *Parp7*^*H532A/H532A*^ mice (*n* = 5). **(I)** Western blot analysis of PARP7 in whole tissue lysates from the lung, liver, kidney, heart, spleen, and brain. **(J)** Western blot analysis of PARP7 in whole lung lysate from WT and *Trex1*^*−/−*^ littermate control mice. Data are shown as mean ± SD. P values were determined by one-way ANOVA (C, G, and H). *P < 0.05, **P < 0.01, ***P < 0.001, ****P < 0.0001, and ns, not significant. Data are representative of three independent experiments. Source data are available for this figure: SourceData F5.

Next, we performed histological analysis of various tissues in WT, *Parp7*^*−/−*^, and *Parp7*^*H532A/H532A*^ mice. *Parp7*^*−/−*^ and *Parp7*^*H532A/H532A*^ mouse liver, kidney, and heart were unremarkable histologically but displayed an elevated ISG signature compared with WT mouse tissues (Fig. S4, A–G). Interestingly, both *Parp7*^*−/−*^ and *Parp7*^*H532A/H532A*^ mice developed perivascular immune cell infiltration in the lung, which is observed by the increased CD45^+^ cells predominantly localizing around blood vessels (Fig. 5, A–C). Analysis of lung tissue lysate revealed increased levels of p-STAT1 and STAT1 in *Parp7*^*−/−*^ and *Parp7*^*H532A/H532A*^ mice (Fig. 5 D). This is consistent with type I interferonopathies, which frequently display lung involvement (Cazzato et al., 2020). Further, immunofluorescent staining of the lung revealed the formation of tertiary lymphoid structures (TLSs), which are comprised of a B220^+^ B cell zone surrounded by CD3^+^ T cells (Fig. 5 E). The formation of TLSs in the lung indicates persistent inflammatory signaling and are observed in some type I interferonopathy models including *Trex1*^*−/−*^ and *Sting*^*V154M/+*^ mice (Randall, 2010; Zhao et al., 2024; Gao et al., 2022, 2024). These data suggest that PARP7 loss-of-function mice develop lung disease like several other type I interferonopathies.

Next, we measured the levels of IgA in the lung because *Parp7*^*−/−*^ and *Parp7*^*H532A/H532A*^ mice had elevated IgA autoantibodies in the serum, and IgA is an important Ig in the mucosa. Both *Parp7*^*−/−*^ and *Parp7*^*H532A/H532A*^ mice had significantly more IgA in the lung compared with WT mice (Fig. 5, F and G). Additionally, we found that *Parp7*^*−/−*^ and *Parp7*^*H532A/H532A*^ mice had significantly elevated levels of serum IgA anti-PL-7 (Fig. 5 H), which is associated with an autoimmune disease called Antisynthetase syndrome, and patients with anti-PL-7 autoantibodies frequently develop severe idiopathic lung disease (Witt et al., 2016). Collectively, these data indicate that elevated IgA may be an important factor for lung disease in *Parp7*^*−/−*^ and *Parp7*^*H532A/H532A*^ mice.

Since the lung was the key organ affected by PARP7 deficiency, we analyzed the protein levels of PARP7 in various mouse tissues and found that PARP7 levels are high in the lung compared with other tissues (Fig. 5 I). Additionally, we compared PARP7 levels in WT and *Trex1*^*−/−*^ mice, which develop a type I interferonopathy due to chronic activation of cGAS-STING signaling by self-DNA (Crow et al., 2006). Levels of PARP7 in the lung are further increased in *Trex1*^*−/−*^ mice compared with WT littermates, which is consistent with PARP7 being an ISG and responding to inflammation in the lung (Fig. 5 J). We also measured ADP-ribosylation using an anti-adenine dinucleotide ribose (ADPr) antibody and various tissue lysates of WT, *Parp7*^*−/−*^ and *Parp7*^*H532A/H532A*^ mice and did not observe a global change (Fig. S4, H–K). Together, these data suggest that PARP7 maintains immune homeostasis in the lung to prevent tissue inflammation and damage.

### PARP7 inhibits IFN-I production by targeting IRF3

**Figure 6.**
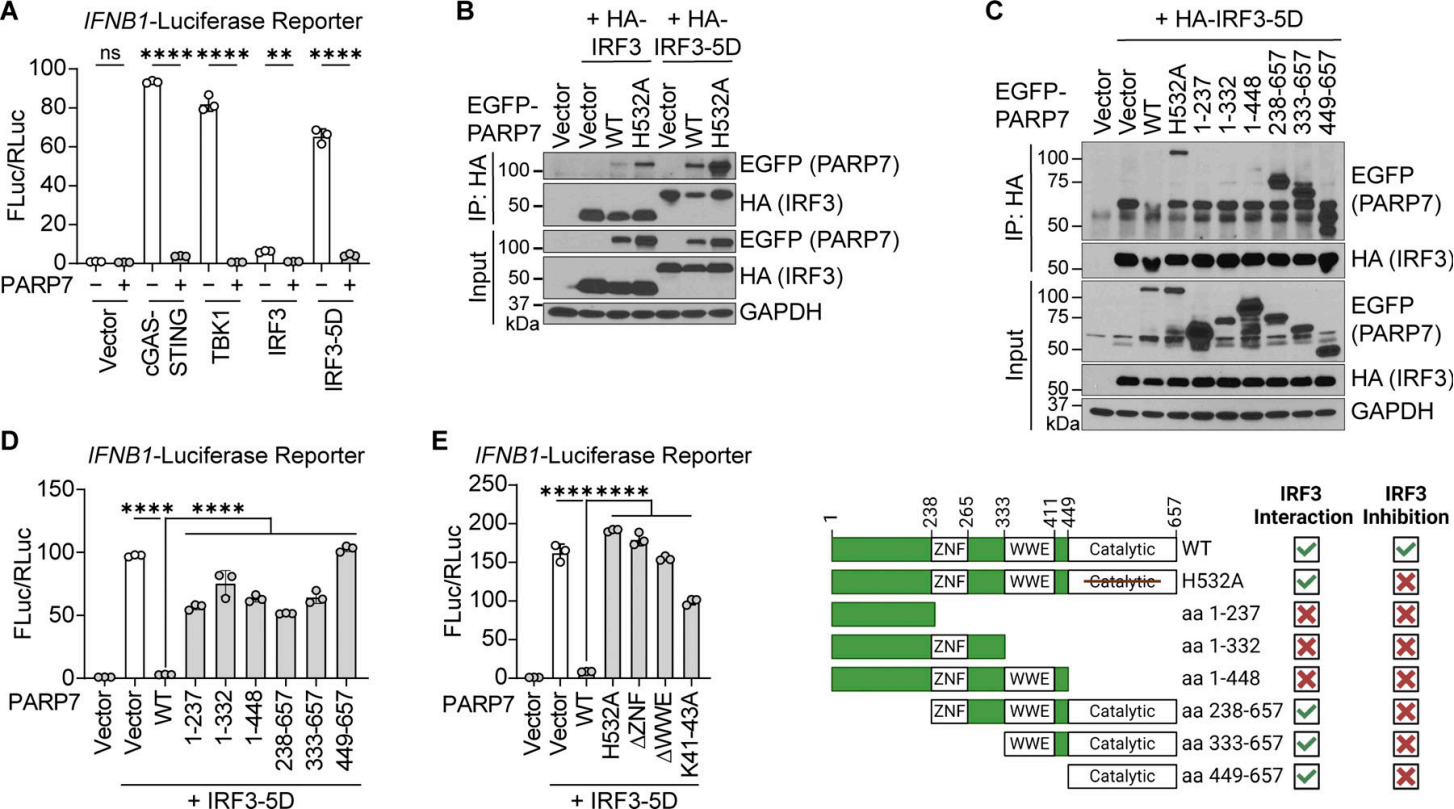
**PARP7 inhibits IFN-I production by targeting IRF3. (A)** Quantification of luciferase activity in HEK293T cells transfected with *IFNB1* firefly luciferase, *TK*-Renilla luciferase, EGFP-PARP7, and other indicated plasmids for 24 h. **(B)** Co-immunoprecipitation analysis of IRF3:PARP7 interaction. HEK293T cells were transfected with HA-IRF3 or HA-IRF3-5D and EGFP-PARP7 or EGFP-PARP7-H532A, followed by IP with HA antibody and blotted with the indicated antibodies. **(C)** Co-immunoprecipitation analysis of IRF3:PARP7 truncation mutants’ interaction. HEK293T cells were transfected with HA-IRF3-5D and EGFP-PARP7 truncation mutants followed, by IP with HA antibody and blotted with indicated antibodies. A schematic diagram shows the PARP7 truncation mutants and summary of mutant activity. **(D)** Quantification of luciferase activity in HEK293T cells transfected with *IFNB1* firefly luciferase, *TK*-Renilla luciferase, HA-IRF3-5D, and various EGFP-PARP7 truncation mutants for 24 h. **(E)** Quantification of luciferase activity in HEK293T cells transfected with *IFNB1* firefly luciferase, *TK*-Renilla luciferase, HA-IRF3-5D, and various EGFP-PARP7 mutants for 24 h. Data are shown as mean ± SD. P values were determined by one-way ANOVA (A, D, and E). *P < 0.05, **P < 0.01, ***P < 0.001, ****P < 0.0001, and ns, not significant. Data are representative of at least three independent experiments. Source data are available for this figure: SourceData F6.

Next, we investigated the mechanism by which PARP7 inhibits IFN-I production. We predicted that PARP7 targets a shared component of innate immune sensing pathways (e.g., TBK1 or IRF3) because PARP7 inhibited IFN-I production downstream of cGAS, RIG-I/MDA5, and TLR4 (Fig. 2). First, we examined the effect of PARP7 on activation of the *IFNB1* promoter induced by different signaling components. PARP7 potently inhibited the activation of the *IFNB1* promoter induced by overexpression of cGAS-STING, TBK1, IRF3, and IRF3-5D, suggesting that PARP7 targets the transcription factor IRF3 (Fig. 6 A). This is consistent with other studies demonstrating that PARP7 acts on transcription factors that induce its expression, including aryl hydrocarbon receptor (MacPherson et al., 2013), estrogen receptor (Rasmussen et al., 2021), androgen receptor (Yang et al., 2021), and hypoxia-inducible factor-1α (Zhang et al., 2020).

Next, we determined whether PARP7 and IRF3 interact using co-immunoprecipitation assays. We found that PARP7 interacts with both IRF3 and IRF3-5D (Fig. 6 B). IRF3-5D constitutively dimerizes and localizes to the nucleus, whereas IRF3 largely remains inactive in the cytoplasm in the absence of innate immune stimulation. Interestingly, PARP7 had a stronger interaction with IRF3-5D compared with IRF3, suggesting that this interaction occurs in the nucleus or requires IRF3 to be in its active conformation. Additionally, IRF3 and IRF3-5D interact with both PARP7 and PARP7-H532A, indicating that the MARylation activity of PARP7 is dispensable for the interaction.

**Figure S5.**
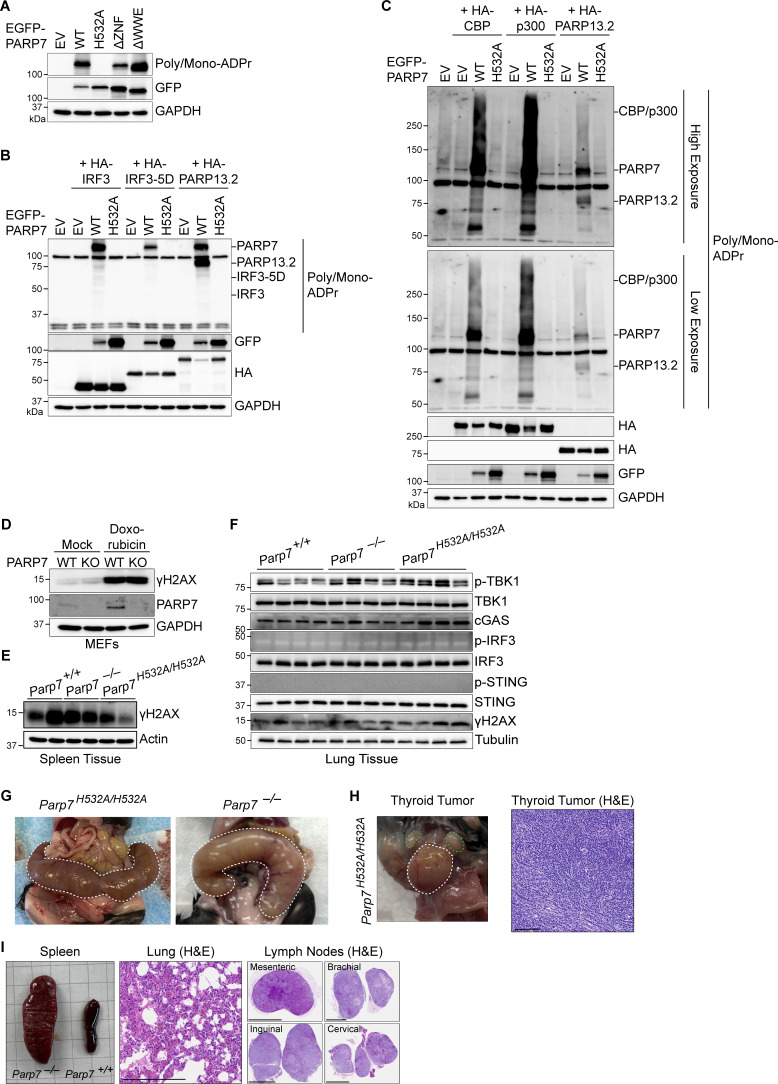
**IRF3 and CBP/p300 MARylation analysis, DNA damage in mouse tissues, and incidental findings in mice. (A)** Western blot analysis of PARP7 auto-MARylation. PARP7 mutants (as indicated on top) were overexpressed in HEK293T cells followed, by blotting with indicated antibodies. **(B)** Western blot analysis of ADP-ribose in whole cell lysates. Empty vector (EV), EGFP-PARP7, or EGFP-PARP7-H532A was co-expressed with either HA-IRF3, HA-IRF3-5D, or HA-PARP13.2 (as indicated on top) in HEK293T cells, followed by blotting with indicated antibodies. **(C)** Western blot analysis of ADP-ribose in whole cell lysates. EV, EGFP-PARP7, or EGFP-PARP7-H532A was co-expressed with either HA-CBP, HA-p300, or HA-PARP13.2 (as indicated on top) in HEK293T, followed by blotting with indicated antibodies. Data are representative of at least three independent experiments. **(D)** Western blot analysis of DNA damage. WT and *Parp7*^*−/−*^ MEFs were treated with mock or doxorubicin (1 μM, 6 h), followed by analysis for γH2AX. **(E)** Western blot analysis of γH2AX in the spleen from WT, *Parp7*^*−/−*^, and *Parp7*^*H532A/H532A*^ mice (*n* = 2). **(F)** Western blot analysis of cGAS–STING pathway components and DNA damage in the lung from WT, *Parp7*^*−/−*^, and *Parp7*^*H532A/H532A*^ mice (*n* = 4). Data are representative of two independent experiments. **(G)** Photo of pyometra (outlined in white dashed line) of 10-mo-old female *Parp7*^*H532A/H532A*^ and *Parp7*^*−/−*^ mouse. **(H)** Photo of noninvasive thyroid tumor (outlined in white dashed lines) and cervical lymph nodes (outlined in green dashed lines) of a 10-mo-old female *Parp7*^*H532A/H532A*^ mouse. H&E staining of the thyroid tumor. Scale bar, 100 μm. **(I)** Photo of spleen of 10-mo-old *Parp7*^*−/−*^ mouse compared with WT littermate mouse. H&E staining of the lung and lymph nodes from *Parp7*^*−/−*^ mouse. Scale bars, 250 μm. Pathology is suggestive of lymphoma for H and I. Source data are available for this figure: SourceData FS5.

We mapped the region of PARP7 that mediates its interaction with IRF3. PARP7 contains an N-terminal nuclear localization sequence (NLS), a CCCH-type zinc finger (ZNF) domain, a tryptophan–tryptophan-glutamate (WWE) domain, and a PARP catalytic domain. We found that truncations of PARP7 containing the catalytic domain were able to interact with IRF3, suggesting that the catalytic domain of PARP7 is necessary and sufficient for the interaction with IRF3 (Fig. 6 C). However, none of the truncation mutants were able to inhibit the activation of the *IFNB1* promoter, indicating that the PARP7–IRF3 interaction is not sufficient for inhibiting IFN-I production (Fig. 6 D). To tease out the requirement for specific domains, we generated individual domain deletions or mutations, including PARP7-H532A, PARP7-ΔZNF, and PARP7-ΔWWE and found that none can inhibit the activation of the *IFNB1* promoter (Fig. 6 E). This was not due to loss of MARylation activity as both PARP7-ΔZNF and PARP7-ΔWWE retain their auto-MARylation activity (Fig. S5 A). Additionally, we tested whether the nuclear localization of PARP7 is required for inhibiting IFN-I production. Mutation of the NLS of PARP7 (K41-43A) rendered it unable to inhibit the activation of the *IFNB1* promoter, suggesting that the nuclear localization of PARP7 is necessary for inhibiting IFN-I production (Fig. 6 E). Collectively, these data suggest that PARP7 binding to IRF3 in the nucleus through its catalytic domain is a key component of the inhibitory mechanism; however, other domains of PARP7 are also required likely by mediating interactions with other binding partners.

### PARP7 inhibits the interaction between IRF3 and the transcriptional co-activators CBP/p300

**Figure 7.**
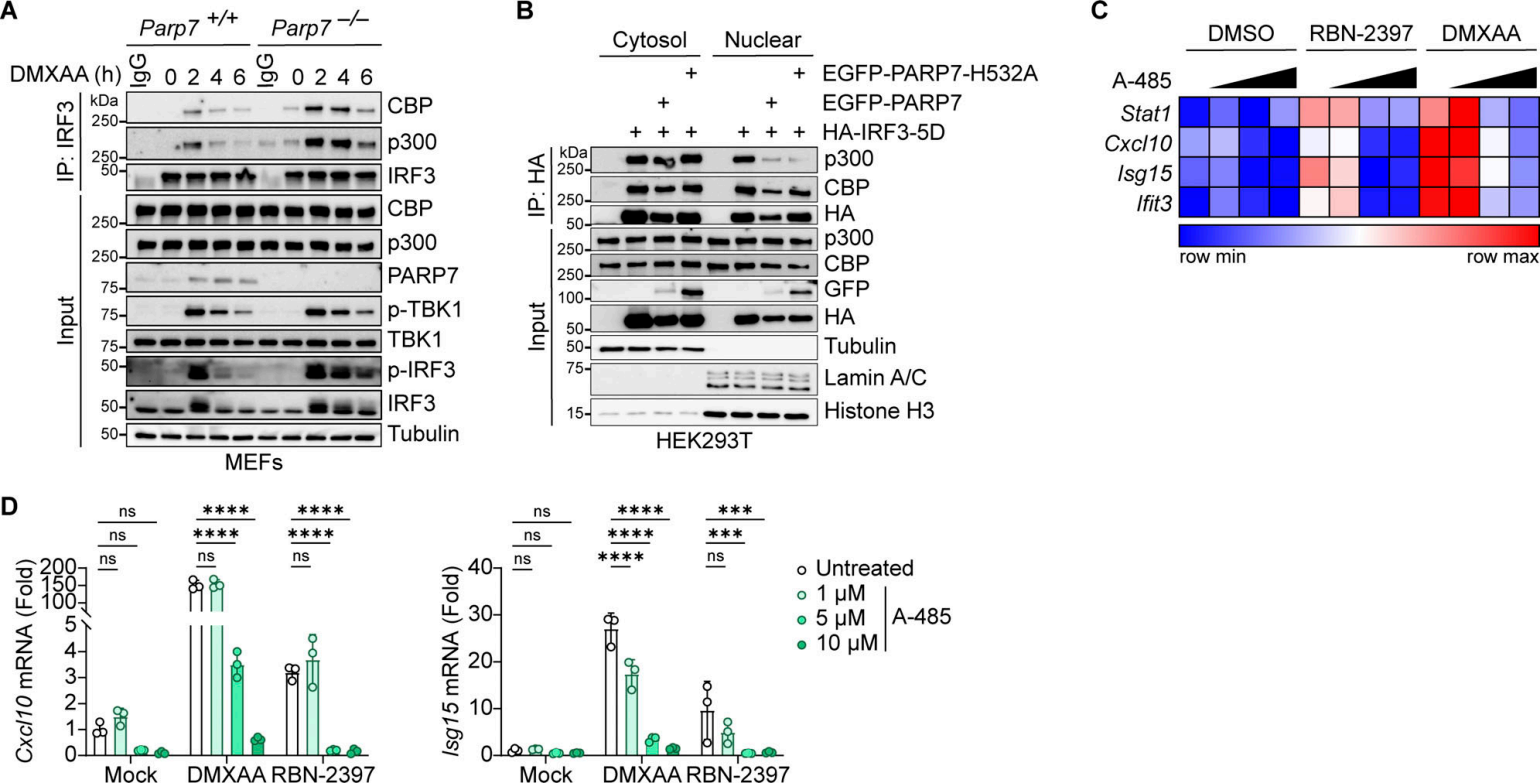
**PARP7 inhibits the interaction between IRF3 and transcriptional co-activators CBP/p300. (A)** Co-immunoprecipitation analysis of endogenous IRF3:CBP/p300 interaction. WT and *Parp7*^*−/−*^ MEFs were stimulated with DMXAA (10 μg/ml) for the indicated time points, followed by IP with IRF3 antibody and blotted with the indicated antibodies. **(B)** Co-immunoprecipitation analysis of IRF3:CBP/p300 interaction. HEK293T cells were transfected with HA-IRF3-5D and EGFP-PARP7 or EGFP-PARP7-H532A, followed by IP with HA antibody and blotted with indicated antibodies. **(C)** A heat map showing the expression of various ISGs following treatment with increasing doses of A-485 (0, 1, 5, and 10 μM, 24 h), DMXAA (10 μg/ml, 4 h), or RBN-2397 (1 μM, 24 h). **(D)** Representative bar graphs of individual ISGs from D. Data are shown as mean ± SD. P values were determined by two-way ANOVA (E). *P < 0.05, **P < 0.01, ***P < 0.001, ****P < 0.0001, and ns, not significant. Data are representative of three independent experiments. Source data are available for this figure: SourceData F7.

Since PARP7 MARylation activity is required for its inhibition of IFN-I production, we examined whether PARP7 MARylates IRF3. However, we did not detect MARylation of IRF3 or IRF3-5D when co-expressed with PARP7 while PARP7 auto-MARylation and PARP13 MARylation (a previously identified target of PARP7 [Rodriguez et al., 2021]) were detected (Fig. S5 B). This suggests that PARP7 binds to but does not MARylate IRF3. Following its phosphorylation by TBK1, IRF3 dimerizes and translocates to the nucleus where it associates with the transcriptional co-activators CBP/p300 to form the active transcriptional holocomplex that binds the promoter of target genes (Schwanke et al., 2020). A previous chemical genetic screen identified CBP/p300 as interactors and targets of PARP7 (Rodriguez et al., 2021). This led us to hypothesize that PARP7 may be recruited to IRF3 and disrupt the transcriptional holocomplex to inhibit IFN-I production. We observed increased interaction between IRF3 and CBP/p300 in *Parp7*^*−/−*^ MEFs stimulated with DMXAA compared with WT MEFs, suggesting that the transcriptional holocomplex persists in the absence of PARP7 (Fig. 7 A). Additionally, PARP7 overexpression reduced the interaction between ectopically expressed IRF3-5D and endogenous CBP/p300 in the nucleus (Fig. 7 B). However, we did not observe MARylation of CBP or p300 when co-expressed with PARP7 in HEK293T cells and blotted with anti-ADPr antibody, while PARP7 auto-MARylation and PARP13.2 MARylation was observed (Fig. S5 C). Together, these data indicate that PARP7 inhibits the production of IFN-I by disrupting the interaction between IRF3 and CBP/p300, leading to a resolution of the transcriptional holocomplex. The exact target of PARP7 remains to be defined.

To further assess the role of CBP/p300 on IFN-I signaling due to inhibition of PARP7, we treated MEFs with RBN-2397 in the presence or absence of a CBP/p300 inhibitor (A-485). RBN-2397 treatment induced the expression of multiple ISGs, which was repressed by A-485 in a dose-dependent manner (Fig. 7, C and D). As a positive control, DMXAA-induced ISG expression was similarly inhibited by A-485. Taken together with our earlier observation of extended p-IRF3 levels in PARP7 knockout and enzymatic-dead mutant cells, these data suggest that PARP7 resolves IRF3 transcriptional activity by dissociating the transcriptional holocomplex. In the absence of PARP7, the IRF3 transcriptional holocomplex fails to dissociate, leading to excessive IFN-I production (Fig. S5 D).

### PARP7 loss-of-function autoimmune disease is dependent on IRF3 transcriptional activity

**Figure 8.**
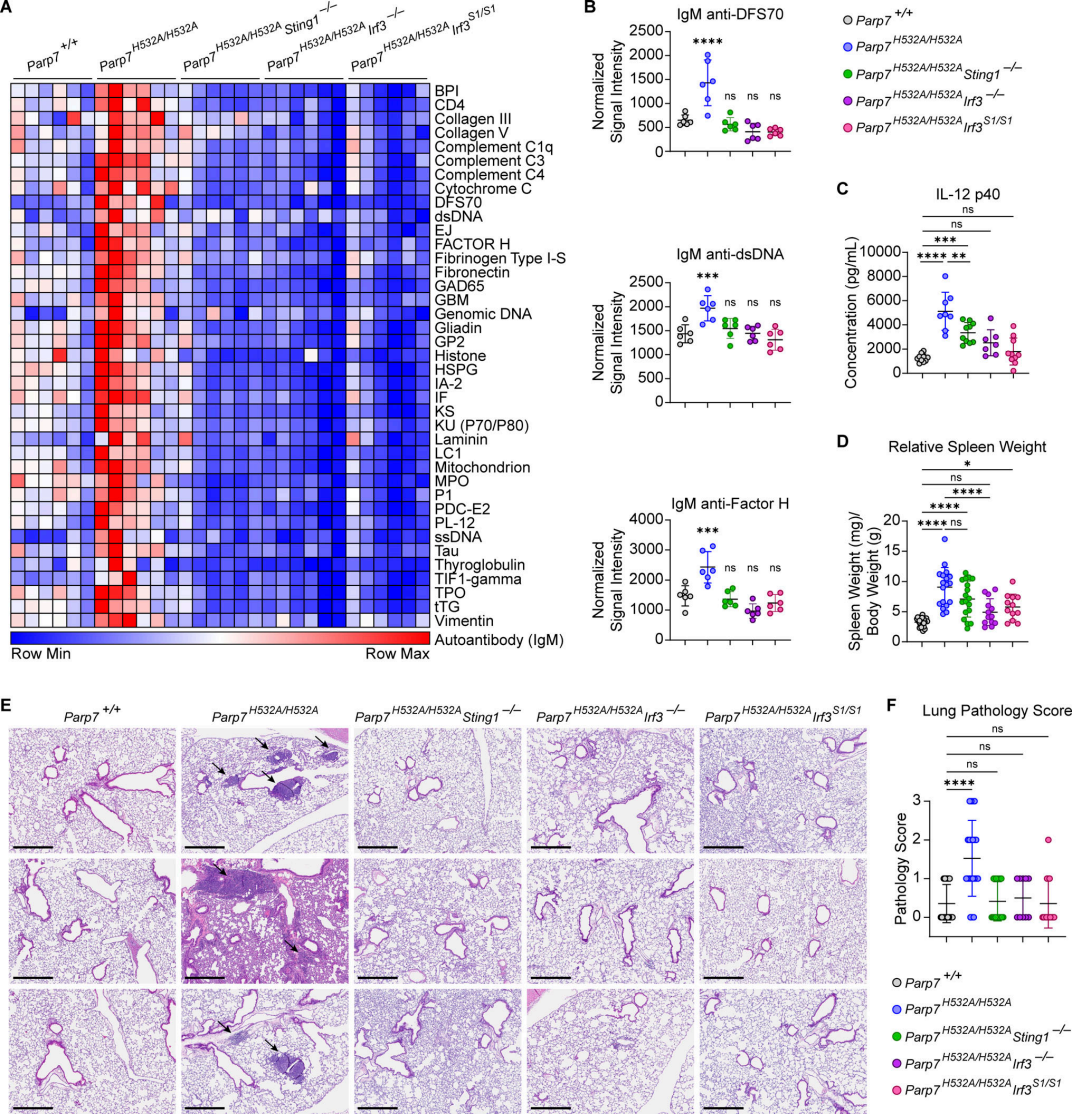
***Parp7*** ^***H532A/H532A***^ **autoimmune phenotype is dependent on IRF3 transcriptional activity. (A)** A heat map showing IgM autoantibody array analysis of mouse serum from 10-mo-old WT, *Parp7*^*H532A/H532A*^, *Parp7*^*H532A/H532A*^*Sting1*^*−/−*^, *Parp7*^*H532A/H532A*^*Irf3*^*−/−*^, and *Parp7*^*H532A/H532A*^*Irf3*^*S1/S1*^ mice (*n* = 6). **(B)** Representative bar graphs of individual IgM autoantibodies from A. **(C)** Bar graph of serum IL-12 p40 from 10-mo-old WT (*n* = 10), *Parp7*^*H532A/H532A*^ (*n* = 8), *Parp7*^*H532A/H532A*^*Sting1*^*−/−*^ (*n* = 10), *Parp7*^*H532A/H532A*^*Irf3*^*−/−*^ (*n* = 7), and *Parp7*^*H532A/H532A*^*Irf3*^*S1/S1*^ (*n* = 10) mice. **(D)** Bar graph showing the relative spleen weight of 10-mo-old WT (*n* = 29), *Parp7*^*H532A/H532A*^ (*n* = 18), *Parp7*^*H532A/H532A*^*Sting1*^*−/−*^ (*n* = 18), *Parp7*^*H532A/H532A*^*Irf3*^*−/−*^ (*n* = 13), and *Parp7*^*H532A/H532A*^*Irf3*^*S1/S1*^ (*n* = 12) mice. **(E)** Representative H&E staining of the lung from 10-mo-old WT, *Parp7*^*H532A/H532A*^, *Parp7*^*H532A/H532A*^*Sting1*^*−/−*^, *Parp7*^*H532A/H532A*^*Irf3*^*−/−*^, and *Parp7*^*H532A/H532A*^*Irf3*^*S1/S1*^ mice. Black arrows denote immune aggregates in lung tissue. Scale bars, 500 μm. **(F)** Graph of lung pathology score based on the lung histology of 10-mo-old WT (*n* = 17), *Parp7*^*H532A/H532A*^ (*n* = 21), *Parp7*^*H532A/H532A*^*Sting1*^*−/−*^ (*n* = 17), *Parp7*^*H532A/H532A*^*Irf3*^*−/−*^ (*n* = 10), and *Parp7*^*H532A/H532A*^*Irf3*^*S1/S1*^ (*n* = 14) mice. Data are shown as mean ± SD. P values were determined by one-way ANOVA (B–D and F). *P < 0.05, **P < 0.01, ***P < 0.001, ****P < 0.0001, and ns, not significant. Data are pooled from at least two independent experiments.

Lastly, we investigated whether the autoimmune phenotype observed in *Parp7*^*H532A/H532A*^ mice was dependent on IRF3 and its transcriptional activity by crossing the *Parp7*^*H532A/H532A*^ mice to *Irf3*^*−/−*^ and *Irf3*^*S1/S1*^ mice. *Irf3*^*S1/S1*^ mice express a mutant IRF3 (S388A/S390A) that lacks the phosphorylation sites required for the transcriptional activity of IRF3; this mutant retains IRF3 cytoplasmic function such as IRF3-mediated pathway of apoptosis (Chattopadhyay et al., 2016). Additionally, we crossed the *Parp7*^*H532A/H532A*^ mice to *Sting*^*–/–*^ mice because the cGAS–STING pathway is a major driver of tonic IFN-I signaling, and both *Sting*^*V154M/+*^ mice (STING gain-of-function) and *Trex1*^*−/−*^ mice (accumulation of self-DNA that activates cGAS-STING) develop a similar lung phenotype, including TLSs (Gao et al., 2022; Zhao et al., 2024). We found that *Parp7*^*H532A/H532A*^*Irf3*^*−/−*^ and *Parp7*^*H532A/H532A*^*Irf3*^*S1/S1*^ mice fully rescued all immunopathology in *Parp7*^*H532A/H532A*^ mice, including increased autoantibodies, inflammatory cytokines, splenomegaly, and perivascular inflammation in the lung (Fig. 8, A–F). In contrast, *Parp7*^*H532A/H532A*^*Sting1*^*−/−*^ mice fully rescued the perivascular inflammation in the lung but only partially rescued other features of autoimmunity. Of note, we did not detect increased DNA damage in PARP7-deficient spleen tissue lysates or PARP7-deficient MEFs treated with mock or doxorubicin (Fig. S5, D and E). We also did not detect STING phosphorylation in lung tissue lysate of PARP7-deficient mice (Fig. S5 F). Together, these data suggest that the systemic autoimmunity and lung disease caused by the loss of PARP7 activity is driven by IRF3 transcriptional activity. Multiple innate sensing pathways likely lead to tonic IRF3 activation in peripheral tissues that are amplified by PARP7 deficiency, although our data suggest that STING is a key driver for lung disease. Further, PARP7 deficiency likely increases homeostatic STING signaling rather than incurring DNA damage that would activate cGAS-STING.

Additionally, we observed several low frequency events in *Parp7*^*−/−*^ and *Parp7*^*H532A/H532A*^ mice (none of which were observed in WT littermate mice). We found that four female *Parp7*^*H532A/H532A*^ or *Parp7*^*−/−*^ mice displayed a pathological phenotype involving their reproductive organs, including three with pyometra and one with an ovarian cyst (Fig. S5 G). This is consistent with a previous study that reported *Parp7*^*−/−*^ mice to be sterile and develop ovarian cysts (Schmahl et al., 2008). Additionally, we found two mice with tumors at 10 mo old. A female *Parp7*^*H532A/H532A*^ mouse had a noninvasive thyroid tumor, and a female *Parp7*^*−/−*^ mouse was found to have metastatic tumors in several tissues (Fig. S5, H and I). Pathology suggests these tumors are lymphoma. Overall, these incidental findings highlight other potential pathologies related to PARP7 loss-of-function, which should be considered during clinical use of PARP7 inhibitors.

## Discussion

ADP-ribosylation is a fundamental posttranslational modification in all organisms. PARP-mediated ADP-ribosylation plays an important role in various cellular functions including innate immunity (Zhu and Zheng, 2021). Here, we screened the entire PARP family to assess their expression and function in IFN-I signaling. We found that many PARPs are ISGs, particularly MARylating PARPs, of which eight PARPs inhibited IFN-β production to varying degrees.

We found that PARP7 was the most potent negative feedback regulator of IFN-I production. Previous studies have implicated PARP7 in IFN-I signaling using PARP7 inhibitors in cancer cells, although the mechanism remained unclear with TBK1 and NF-κB suggested as potential targets (Yamada et al., 2016; Rasmussen et al., 2023). Here, we show that PARP7 is induced by IFN-I signaling, then binds to IRF3 and disrupts the IRF3:CBP/p300 transcriptional holocomplex, thereby terminating IFN-I production. This mechanism forms a rapid negative feedback loop that dials the scale of IFN-I production and, consequently, its downstream responses. This function requires the MARylation activity of PARP7, although we did not observe MARylation of IRF3 or CBP or p300 when co-expressed with PARP7. Both CBP and p300 were identified as interactors and targets of PARP7 in a previous chemical genetic screen using a PARP7-specific chemical labeling tool (Rodriguez et al., 2021); thus, it remains a possibility that PARP7 modifies CBP/p300 at low levels or transiently that cannot be detected by conventional anti-ADPr blotting.

The PARP7 mechanism is distinct from the few known negative feedback ISGs, such as ISG15 and USP18, which function downstream of IFN-I production to quell IFNAR-JAK-STAT signaling (Meuwissen et al., 2016; Zhang et al., 2015). In comparison, PARP7 acts upstream of IFN-I production by inhibiting its transcription. Negative feedback ISGs are essential in limiting the tissue toxicity of IFN-I. Humans deficient in USP18 or ISG15 develop severe autoimmune disease due to uncontrolled IFN-I signaling (Zhang et al., 2015; Meuwissen et al., 2016; Bogunovic et al., 2012; Martin-Fernandez et al., 2020; Malakhova et al., 2006). We show that PARP7 loss-of-function mice also develop systemic autoimmunity that is dependent on IRF3. IRF3 has multiple activities both in the cytoplasm and in the nucleus. IRF3 ubiquitination in the cytoplasm induces apoptosis through the IRF3-mediated pathway of apoptosis pathway (Chattopadhyay et al., 2016). IRF3 phosphorylation and transcriptional activity in the nucleus induce expression of IFN/ISGs and pro-apoptotic genes (Manetsch et al., 2023). The IRF-S1 mouse used in this study selectively disrupts its transcriptional activity in the nucleus, and it fully rescued *Parp7*^*H532A/H532A*^ mouse disease. Therefore, we believe the systemic autoimmunity due to PARP7 deficiency requires IRF3 transcriptional activity.

Although pathogenic *PARP7* mutations have not been reported in humans, PARP7 inhibitors were recently tested in clinical trials (NCT04053673 and NCT05127590), but early indications of anticancer results were not impressive. We showed here in human THP-1 monocytes and human healthy donor PBMCs that PARP7 inhibition led to increased IFN-I signaling. Therefore, our study provides the first comprehensive analysis of immune pathology associated with PARP7 loss-of-function that should guide clinical practice when evaluating immune-associated adverse events in patients treated with PARP7 inhibitors.

*Parp7* ^*H532A/H532A*^ mice developed more severe autoimmunity than *Parp7*^*−/−*^ mice. The reason is unclear, although we can speculate two possibilities. One possibility is “substrate trapping”—we showed that PARP7-H532A binds to IRF3 stronger than PARP7-WT; therefore, it is possible that this enzymatic-dead PARP7 mutant “holds” the IRF3 transcriptional complex on the DNA, thereby further boosting IFN production, while a deficiency in PARP7 allows the transcriptional complex to stay longer than WT, but the complex will eventually “fall off.” Another possibility is that PARP7 may have an unknown MARylation-independent function that is enhanced due to the increased protein level of PARP7-H532A in the *Parp7*^*H532A/H532A*^ mouse compared with endogenous PARP7 level in the WT mouse. Future studies are needed to investigate these possibilities further.

Our rescue data suggest that the STING pathway is a key signal that drives autoimmunity and lung disease in *Parp7*-deficient mice. Other innate immune pathways also play roles in systemic inflammation because *Sting*^*−/−*^ did not fully rescue serum cytokine levels and splenomegaly. Both *Parp7*^*−/−*^ and *Parp7*^*H532A/H532A*^ mice develop TLSs in the lung as distinctive immunopathology that was dependent on STING or IRF3. Two other mouse models, *Trex1*^*−/−*^ and *Sting*^*V154M/+*^, also show TLS in the lung; both are STING-dependent, but each requires a distinct mechanism (Gao et al., 2022; Zhao et al., 2024). *Trex1*^*−/−*^ leads to self-DNA accumulation, and mouse disease requires cGAS, STING, IRF3, and IFNAR (Stetson et al., 2008; Gall et al., 2012). *Sting*^*V154M/+*^ leads to STING gain-of-function, and mouse disease does not require IRF3 or IFNAR (Motwani et al., 2019). Further studies are necessary to define the cell type(s) responsible for the lung pathology in PARP7 loss-of-function mice and dependency on the IFN-I receptor. In the tumor setting, TLSs promote the influx of immune cells into the tumor microenvironment and correlate with better prognosis and clinical outcome particularly with immunotherapy (Rodriguez and Engelhard, 2020). Therefore, given the abundance of PARP7 in the lung and its natural immunosuppressive function, PARP7 inhibition may be especially effective for treating lung cancer.

PARP7 targeting of the IRF3:CBP/p300 transcriptional holocomplex could be harnessed to treat other autoimmune diseases such as systemic lupus erythematosus (SLE). IFN-I signature is the most recognized gene signature in SLE, and drugs targeting downstream signaling components such as IFNAR or JAK-STAT are being tested in clinical trials with limited benefit (Richter et al., 2022). Targeting transcription factors like IRF3 is difficult, and targeting CBP/p300 may affect other transcription factors. The PARP7 mechanism described here offers new opportunities to harness the natural negative feedback loop to limit IFN-I production irrespective of spontaneous stimuli, which is beneficial for heterogenous autoimmune diseases like SLE. In summary, our study uncovers PARP7 as a negative feedback regulator of IFN-I production that may have many therapeutic implications in autoimmune disease and cancer.

## Materials and methods

### Mice

Animal work was approved by the Institutional Animal Care and Use Committee at University of Texas Southwestern (UTSW) Medical Center. WT (*C57BL/6J*), *Ifnar1*^*−/−*^, and *Trex1*^*−/−*^ mice were purchased from Jackson Laboratory (#030097, #028288, #034907). *Sting1*^*−/−*^ were provided by Glen Barber (University of Miami, Miami, FL, USA). *Irf3*^*−/−*^ mice were provided by Kate Fitzgerald (University of Massachusetts, Boston, MA, USA) with permission from T. Taniguchi (University of Tokyo). *Irf3*^*S1/S1*^ mice were provided by Ganes Sen (Lerner Research Institute, Cleveland Clinic, Cleveland, OH, USA). *Parp7*^*−/−*^ and *Parp7*^*H532A/H532A*^ mice were provided by Jason Matthews (University of Olso, Oslo, Norway). Both male and female mice were used for experiments. All mice were housed in a standard pathogen-free barrier facility under 12 h light-dark cycles. For in vivo cGAMP challenge, 8–10-wk-old mice were injected i.p. with 10 mg/kg 2′,3′-cGAMP (tlrl-nacga23; Invivogen). Blood was collected at indicated time points into a capillary blood collection tube (16.440.100; Sarstedt) and incubated on ice for 30 min, followed by centrifugation at >18,000 rcf for 15 min. Serum was collected from the tubes and stored at −80°C until further analysis.

### Cell lines

HEK293T and THP-1 were obtained from ATCC (CRL-3216, TIB-202). HEK293T and MEF cells were cultured in DMEM (D5617; Sigma-Aldrich) with 10% (vol/vol) FBS, 10 mM HEPES, 2 mM L-glutamine, and 1 mM sodium pyruvate with the addition of 100 U/ml penicillin and 100 mg/ml streptomycin, at 37°C with 5% CO_2_. THP-1 and PBMCs were cultured in RMPI with 10% (vol/vol) FBS, 10 mM HEPES, 2 mM L-glutamine, and 1 mM sodium pyruvate with the addition of 100 U/ml penicillin and 100 mg/ml streptomycin, at 37°C with 5% CO_2_. Mycoplasma tests were conducted monthly and confirmed to be negative.

### Reagents

Immune agonists used include HT-DNA (D6898; Sigma-Aldrich), DMXAA (tlrl-dmx; Invivogen), diABZI (S8796; Selleck Chemical), Poly(I:C) HMW (tlrl-pic; Invivogen), LPS-B5 (tlrl-pb5lps; Invivogen), and recombinant murine IFN-β (8234-MB; R&D Systems). HT-DNA and Poly(I:C) were transfected into cells using Lipofectamine 2000 (11668019; Thermo Fisher Scientific) in Opti-MEM (31985070; Thermo Fisher Scientific). PARP7 inhibitor used was RBN-2397 (HY-136174; MedChemExpress). CBP/p300 inhibitor used was A-485 (HY-107455; MedChemExpress). Proteasome inhibitor used was MG-132 (S2619; Selleck Chemicals). Concentration used and duration of stimulations are noted in figure legends.

### Generation of CRISPR/Cas9 knockout cells

The LentiCRISPRV2 (#52961; Addgene) plasmid was used to generate knockout cell lines. Target guide sequences include mParp7: 5′-GAC​TGT​GTA​GTA​CAG​CCT​CC-3′, 5′-CTG​GAA​ATC​AAC​CCA​TCG​TG-3′ and hPARP7: 5′-CCG​AAC​CTG​AGC​CAG​ACT​GT-3′, 5′-GAC​TGT​GTA​GTG​CAG​CCT​CC-3′. Lentiviruses carrying the sgRNA and Cas9 were generated in HEK293T with the packaging plasmid psPAX2 (#12260; Addgene) and the envelope plasmid pMD2.G (#12259; Addgene). Cells were selected with antibiotics after transduction for several days. Single-cell clones were selected and verified by western blot or sequencing for CRISPR-Cas9 knockout cell lines.

### Generation and immortalization of MEFs

*Parp7* ^*+/−*^ breeding pair was set up to generate littermate paired WT and *Parp7*^*−/−*^ primary MEFs. Embryos were harvested between E11–E13. The head and fetal liver were removed from the embryo and used for genotyping. Embryo was minced with a sterile razor blade in 1 ml of trypsin and transferred to a 15-ml conical. 20 U DNase was added to minced embryo and incubated in a 37°C water bath for 10 min with frequent vortexing to dissociate the cells. Trypsin was quenched with 10 ml of DMEM (D5617; Sigma-Aldrich) with 20% (vol/vol) FBS, 10 mM HEPES, 2 mM L-glutamine, and 1 mM sodium pyruvate with the addition of 100 U/ml penicillin and 100 mg/ml streptomycin (Complete D20). Cell suspension was passed through a 70-μm strainer and distributed on a plate. Primary MEFs were cultured in Complete D20 at 37°C with 5% CO_2_. Primary MEFs were immortalized with SV40 large T antigen using the pBABE neo large T genomic plasmid (#1780; Addgene). Retroviruses were generated in HEK293T with the packaging plasmid pUMVC (#8449; Addgene) and the envelope plasmid VSVG. Cells were selected with antibiotics after transduction for several days and immortalization was confirmed by western blot for SV40 large T (15729; CST).

### Generation of BMDMs

Bone marrow was isolated from the mouse tibia and femur following aseptic technique. 1x RBC Lysis Buffer (420302; BioLegend) was added to the bone marrow for 1 min at room temperature. The lysis reaction was neutralized by fourfold volume of DMEM with 10% (vol/vol) FBS. Bone marrow cells were pelleted and plated in 15-cm dish in DMEM with 10% (vol/vol) FBS and 20% (vol/vol) L929 cell-conditioned media for differentiation into BMDMs. 20 ml of fresh media was added to the dish on day 3. Differentiated BMDMs were collected on day 6 for analysis.

### Isolation of human PBMCs

PBMCs were isolated according to the SepMate (85450; Stem Cell Technologies) protocol using Lymphoprep (07801; Stem Cell Technologies). In brief, buffy coat was diluted 1:1 with PBS and underlaid with Lymphoprep in SepMate tubes. Cells were centrifuged at 1,200 × *g* for 10 min at room temperature, and the top layer containing enriched PBMCs was poured off. PBMCs were washed with PBS with 2% FBS, followed by RBC lysis (420302; BioLegend) and another wash. PMBCs were frozen down and stored in liquid nitrogen until use.

### Dual luciferase reporter assay

HEK293T cells seeded in a 12-well plate were transiently transfected with 400 ng IFNB1-Firefly Luc and 20 ng TK-Renilla Luc together with 400 ng of various expression plasmids and/or empty vector controls using Lipofectamine 2000 (11668019; Thermo Fisher Scientific) in Opti-MEM (31985070; Thermo Fisher Scientific). 24 h later, cells were lysed in Passive Lysis Buffer (E194A; Promega) and centrifuged for 20,000 × *g* for 15 min at 4°C. Equal portions of the lysate were subjected to dual luciferase assay (E1910; Promega) following the manufacturer’s instructions using a microplate reader (Synergy; BioTek) with Gen 5 software (Gen5; BioTek).

### siRNA knockdown

Predesigned siRNA oligomers were purchased from Sigma-Aldrich and dissolved in water to 20 μM. siRNA was reverse transfected using Lipofectamine RNAiMAX reagent (13778150; Thermo Fisher Scientific) in Opti-MEM media (51985034; Thermo Fisher Scientific). siRNAs used in this study include the following: simIrf3 #1 (SASI_Mm02_00323626), simIrf3 #2 (SASI_Mm01_00143487), and siLuciferase (5′-CAU​UCU​AUC​CUC​UAG​AGG​AUG-3′), which was used as a negative control.

### Co-immunoprecipitation assay

Cells were washed once with PBS buffer, lysed in IP Lysis Buffer (20 mM Tris-HCl, pH 7.4, 0.5% Nonidet-P40, 150 mM NaCl, 1x protease inhibitor cocktail [11836170001; Roche], 1x phosphatase inhibitor cocktail [PHOSS-RO; Roche], and 1 μM veliparib [S1004; Selleck Chemicals]) and centrifuged at 20,000 × *g* for 20 min at 4°C. In all, 5% of the supernatants were saved as input. In total, 25 μl of the Protein G Dynabeads (10004D; Thermo Fisher Scientific) or Protein A Dynabeads (10001D; Thermo Fisher Scientific) were incubated with primary antibody for 2 h with rotation at room temperature. Antibodies used for immunoprecipitation include HA (sc-7392; Santa Cruz) and IRF3 (4302; CST). Protein concentration was determined by BCA assay (23225; Thermo Fisher Scientific), and equal amounts of protein were incubated with antibody-bound beads with rotation overnight at 4°C. Beads were washed three times with IP buffer. IP complex was eluted in 1x Western Blot Sample Buffer (EC887, 5x; National Diagnostics) and boiled at 95°C for 5 min. 5x Protein Loading Buffer from National Diagnostics contains 1.0 M Tris-HCl (pH 8.5), 8% (wt/vol) lithium dodecyl sulfate, 40% (vol/wt) glycerol, 2 mM EDTA, 0.5 M DTT, and tracking dye in distilled/deionized water. 1x western blot sample buffer was made from dilution of 5x protein loading buffer with IP lysis buffer.

### Western blot analysis

Equal amounts of protein were separated by SDS-PAGE, followed by transferring to nitrocellulose membrane. For Native-PAGE, equal amounts of protein were prepared in 5x Native-PAGE loading buffer (0.3 mM Tris-HCl, pH 6.8, 50% glycerol, and 0.01% bromophenol blue) and separated on a non-denaturing resolving gel run in inner buffer (25 mM Tris-HCl, pH 8.4, 192 mM glycine, and 1% sodium deoxycholate) and outer buffer (25 mM Tris-HCl, pH 8.4 and 192 mM glycine). Gel was washed in SDS running buffer for 30 min to remove sodium deoxycholate, followed by transferring to nitrocellulose membrane. Membrane blots were blocked in 5% milk in TBST buffer for 1 h at room temperature, followed by incubation with primary antibodies in 3% BSA in TBST buffer at 4°C overnight. After several washes, membrane blots were incubated with HRP-conjugated IgG secondary antibody for 1 h at room temperature. Then, membrane blots were developed with SuperSignal West Pico Chemiluminescent Substrate (34580; Thermo Fisher Scientific) and blue autoradiography film (BDB810; Dot Scientific) or using the ChemiDoc (Bio-Rad). The films were scanned using LIDE 300 scanner (2995C002; Canon). Primary antibodies used for western blot included phospho-TBK1 (5483; CST), TBK1 (3504; CST), phospho-IRF3 (4947; CST), IRF3 (4302; CST), phospho-STING (72971; CST), STING (13647; CST), phospho-STAT1 (9167; CST), STAT1 (9172; CST), IFN-β (CST; 97450), p300 (57625; CST), CBP (7389; CST), GAPDH (2118; CST), alpha-Tubulin (T5168; Sigma-Aldrich), β-Actin (4967; CST), Lamin A/C (2032; CST), Histone H3 (4499; CST), FLAG (14793; CST), HA (3724; CST), GFP (A11122; Invitrogen), and mono/poly-ADPr (83732; CST). PARP7 antibody was provided by Jason Matthews (University of Oslo). Secondary antibodies used for western blot or dot blot included goat anti-rabbit IgG-HRP conjugate (1706515; Bio-Rad), goat anti-mouse IgG-HRP conjugate (1706516; Bio-Rad), goat anti-mouse IgA alpha chain-HRP conjugate (ab97235; Abcam), goat anti-mouse IgM-HRP conjugate (ab97230; Abcam), and TidyBlot Western Blot Detection Reagent:HRP (STAR209PA; Bio-Rad).

### RNA isolation and quantitative RT-PCR

Total RNA was isolated with TRI reagent (T9424; Sigma-Aldrich) according to the manufacturer’s protocol, and cDNA was synthesized with iScript Reverse Transcription Supermix Kit (1708840; Bio-Rad). Quantitative RT-PCR (qPCR) was performed using iTaq Universal SYBR Green Supermix (1725120; Bio-Rad) and CFX Connect Real-Time Detection System (1855201; Bio-Rad) and Bio-Rad CFX Maestro Software (12013758; Bio-Rad). qPCR primer sequences are listed in the supplementary files (Table S1).

### IFN-β ELISA

For serum, blood was collected and incubated on ice for 30 min to clot, followed by centrifugation at 18,000 rcf for 15 min. Serum or supernatant were stored at −80°C until further analysis. Mouse IFN-β ELISA was performed with the VeriKine-HS Mouse IFN-β Serum ELISA (42410-2; PBL) as per the manufacturer’s instructions. The absorbance of the ELISA plate was measured by a microplate reader (Synergy HT; BioTek) using Gen 5 software (Gen5; BioTek).

### Autoantibody and cytokine array

Mouse serum was isolated by centrifuging clotted blood at 18,000 rcf for 10 min. Serum was immediately frozen at −80°C until further analysis. Mouse serum was subjected to either Bio-Plex Pro Mouse Cytokine 23-plex assay (M60009RDPD; Bio-Rad) for serum cytokine analysis or autoantigen Microarray Super Panel I (UTSW) for autoantibody analysis by the Microarray and Immune Phenotyping Core at UTSW. Heat maps were generated with Morpheus (https://software.broadinstitute.org/morpheus).

### Flow cytometry

Immunophenotyping of mouse tissue was performed on the Beckman-Coulter CytoFLEX and analyzed using FlowJo software. Single-cell suspensions of splenocytes and bone marrow were obtained. Cells were counted, and equal number of cells were stained with TruStain FcX antibody (101320; BioLegend) and live-dead Zombie aqua dye (423102; BioLegend). Antibodies included in the splenic T cell panel include CD3-AF700 (100216; BioLegend), CD4-BV421 (100443; BioLegend), CD8-PE/Cy5 (100710; BioLegend), CD44-APC (103012; BioLegend), and CD62L-PE/Cy7 (104418; BioLegend). Antibodies included in the splenic B cell panel include IgM-FITC (406506; BioLegend), IgD-PE/Cy7 (405720; BioLegend), CD23-BV421 (101621; BioLegend), CD21-PE (12-0212-82; eBioscience), CD19-PerCP/Cy5.5 (152406; BioLegend), and B220-APC (103212; BioLegend). Antibodies included in the bone marrow B cell panel include CD43-BV421 (562958; BD Bioscience), B220-PE (103208; BioLegend), IgD-PE/Cy7 (405720; BioLegend), and IgM-APC/Cy7 (406516; BioLegend). Antibodies included in the spleen myeloid panel include CD11b-APC (101211; BioLegend), Ly6C-PE (128007; BioLegend), Ly6G-PE/Cy7 (127618; BioLegend), CD3-FITC (100204; BioLegend), and CD19-FITC (557398; BD Biosciences).

### Tissue histology, immunohistochemistry, and immunofluorescence

Tissue was isolated from mice after whole body perfusion with PBS. Tissue was fixed in 4% paraformaldehyde (J19943-K2; Thermo Fisher Scientific) for 24 h at 4°C and washed in PBS before being embedded in paraffin blocks. Tissue sections were subjected to H&E staining, immunohistochemistry staining for CD45 (70257; CST) and IgA (ab97231; Abcam), or immunofluorescence staining for CD3 (ab16669; Abcam) and B220 (103204; BioLegend). The secondary antibodies used were goat anti-rabbit IgG Alexa-Fluor 555 (A32794; Thermo Fisher Scientific) and streptavidin Alexa-Fluor 488 (S11223; Thermo Fisher Scientific). Tissue slides were digitally scanned using the Nanozoomer 2.0HT (Hamamatsu Photonics) for bright field or Axioscan Z1 (ZEISS) for fluorescent. Histology was reviewed by a board-certified pathologist. Lung pathology was scored on the following scale: 0, no foci of immune infiltration observed in the lung; 1, a few (1–3) foci of perivascular immune cell aggregates; 2, multiple (≥4) foci of perivascular immune cell aggregates; and 3, continuous perivascular/peribronchial immune cell infiltration that affects at least half of a lobe. Quantification of IgA staining in the lung was performed using QuPath (Bankhead et al., 2017).

### Statistics

Statistical tests were noted in figure legends. All data were shown as means ± SD. Analyses were performed using GraphPad Prism 10 software (GraphPad). Statistical significance was identified with *P < 0.05, **P < 0.01, ***P < 0.001, ****P < 0.0001, and ns, not significant. Schematic graphs were created with https://Biorender.com.

### Online supplemental material

Fig. S1 addresses the effect of PARP7 on NF-κB signaling and the protein stability of PARP7. Fig. S2 shows the Mendelian ratio, weight, IgG autoantibodies, individual autoantibodies, and serum Ig levels in WT, *Parp7*^*−/−*^ and *Parp7*^*H532A/H532A*^ mice. Fig. S3 shows the representative flow plots from Fig. 4, J–L, and flow cytometry analysis of B cell populations in the spleen and bone marrow. Fig. S4 shows the tissue histology, ISG signature, and ADP-ribose levels in various mouse tissues. Fig. S5 addresses whether PARP7 MARylates IRF3, CBP, or p300, and whether PARP7 affects DNA damage levels, and the incidental findings in *Parp7*^*−/−*^ and *Parp7*^*H532A/H532A*^ mice. Table S1 shows the list primer sequences used for qRT-PCR.

## Supplementary Material

Table S1 shows the list of primer sequences used for qPCR.

SourceData F2 is the source file for Fig. 2.

SourceData F3 is the source file for Fig. 3.

SourceData F5 is the source file for Fig. 5.

SourceData F6 is the source file for Fig. 6.

SourceData F7 is the source file for Fig. 7.

SourceData FS4 is the source file for Fig. S4.

SourceData FS5 is the source file for Fig. S5.

## Data Availability

All data associated with this study are available in the paper or the online supplemental material.
