# Supplementary material for: PARP7 inhibits type I interferon signaling to prevent autoimmunity and lung disease
Source: J Exp Med. 2025 Feb 19;222(5):e20241184. doi: 10.1084/jem.20241184 (PMC11837972; doi:10.1084/jem.20241184)
Supplement: Table S1 — shows the list of primer sequences used for qPCR. [file jem_20241184_tables1.docx]

Table S1: List of primer sequences used for qPCR

| **Gene** | **Forward Primer** | **Reverse Primer** |
| --- | --- | --- |
| *Gapdh* | TTCACCACCATGGAGAAGGC | GGCATCGACTGTGGTCATGA |
| *Hprt* | ATGTCATGAAGGAGATGGGAGGCCA | TCTCCACCAATAACTTTTATGTCCC |
| *Parp1* | GGCAGCCTGATGTTGAGGT | GCGTACTCCGCTAAAAAGTCAC |
| *Parp2* | CACAGCTTGGTGACTTGTTCT | ACTCAGGCTTCAAAGTTTCCTC |
| *Parp3* | ATGGCTCCAAAACGAAAGGC | CACGGATGACCCGATTATCAG |
| *Parp4* | TCATACCACCTAAGTTGGGTCC | AGCAAAGCTACCTGAGAAAGC |
| *Parp5a* | GTCTACTCCGTTACACCTGGC | TGAAGAGGTACAAGTCCACCTTT |
| *Parp5b* | CGCCCGAGAAGGTGAACAG | TTTGCACCGTTCTGAAGAAGAT |
| *Parp6* | CTGACCTGTATAGACACCCACA | AAAACCGTAGCCTCAACACAATA |
| *Parp7* | ACATCACACCGTATTGCCCT | GCCCAAAAGTCTTGTCCTCCAT |
| *Parp8* | TAAATCGCACAAACTTTTGGGC | TCTCCAGAACAAGATCGAGTCAA |
| *Parp9* | AGGACGCCAAAGGGATCTG | CCGGCTCCATAAACTGGGT |
| *Parp10* | CTCTGTACTTTGAAAACCACCGT | GCTCAGTCGGACACCATGTA |
| *Parp11* | GTGGACGACATGGACACATCG | CCAGTGACAAGATTCATCTGCTT |
| *Parp12* | TCATCTACGGCAACTGCAAGT | AGCTCAGTATATGTGAGGTGGTC |
| *Parp13* | CCCGAAGCGCAACTCTACG | CGCTGGGACTGTGCATAGTG |
| *Parp14* | AAGCAGATTGAAGTTGAGGACAA | CTTTGCCGGGGTTTCTGAAGT |
| *Parp16* | GCCACGACTGTAAGGACTTCG | CACTGTGGATTGTCAGGATCTTG |
| *Ifnb1* | GTCCTCAACTGCTCTCCACT | CCTGCAACCACCACTCATTC |
| *Stat1* | AAGGTGAAGCCAATGGTGTGGCGAA | CCGATGCAGGCGCTCTGCTGCCTTC |
| *Stat4* | TCAGTGAGAGCCATCTTGGAGG | TGTAGTCTCGCAGGATGTCAGC |
| *Ifit1* | TTCACATGGAAGCTGCTATTTGAAA | TGCTCAGCTGCTCGCTCTGGATCAA |
| *Ifit2* | ATGAGTTTCAGAACAGTGAGTTTAA | AACTGGCCCATGTGATAGTAGACCC |
| *Ifit3* | GAGGTGGCTGAGATGGGGGCACCG | CTCCAGTCACATCACCCACCATCTT |
| *Ifitm3* | CCTATGCCTACTCCGTGAAGTCTA | AGTGTGAAGGTTTTGAGCGTT |
| *Isg15* | TCTGACTGTGAGAGCAAGCAG | ACCTTTAGGTCCCAGGCCATT |
| *Ifi30* | GGATAAGCTGGAAAAGGAGGCAG | TCTGGTGACACCTCAGGAGCAT |
| *Ifi35* | TGCTCTGGTCACCTTTGAGGAC | CTGAATGTTGGTCACCACAGGC |
| *Ifi44* | AACTGACTGCTCGCAATAATGT | GTAACACAGCAATGCCTCTTGT |
| *Oas3* | TCTGGGGTCGCTAAACATCAC | GATGACGAGTTCGACATCGGT |
| *Usp18* | GTGTCCGTGATCTGGTCCTT | CTGCAGAAATACAACGTGCC |
| *Bst2* | CAAACTCCTGCAACCTGACCGT | CTCCTGGTTCAGCTTCGTGACT |
| *Irf7* | TCGCACAGTCTTCCGCGTACCCTGG | TTCCAGCCTCTTCGCTCTCTTCGCT |
| *Cxcl2* | GCGCTGTCAATGCCTGAAGA | TTTGACCGCCCTTGAGAGTG |
| *Cxcl9* | TGTGGAGTTCGAGGAACCCT | TGCCTTGGCTGGTGCTG |
| *Cxcl10* | GGGATCCCTCTCGCAAGGACGGTCC | ACGCTTTCATTAAATTCTTGATGGT |
| *Cxcl11* | CCGAGTAACGGCTGCGACAAAG | CCTGCATTATGAGGCGAGCTTG |
| *Ccl2* | GCATCCACGTGTTGGCTCA | CTCCAGCCTACTCATTGGGATCA |
| *Ccl3* | TGAAACCAGCAGCCTTTGCTC | AGGCATTCAGTTCCAGGTCAGTG |
| *Ccl5* | ACTCCCTGCTGCTTTGCCTAC | ACTTGCTGGTGTAGAAATACT |
| *Ccl8* | AAGCTGACTGGGCCAGATAAGGCTC | CAAGGATCTCCATGTACTCACTGAC |
| *HPRT* | GCACAGAGCCTCGCCTT | GTTGTCGACGACGAGCG |
| *IFNA* | GCTTTACTGATGGTCCTGGTGGTG | GAGATTCTGCTCATTTGTGCCAG |
| *IFNB1* | CAAGTGTCTCCTCCAAATTGCTCTC | TCTCCTCAGGGATGTCAAAGTTCAT |
