# Supplementary figures and images for: PARP7 inhibits type I interferon signaling to prevent autoimmunity and lung disease
Source: J Exp Med. 2025 Feb 19;222(5):e20241184. doi: 10.1084/jem.20241184 (PMC11837972; doi:10.1084/jem.20241184)

SOURCE DATA: FIGURE 2

Figure 2I:

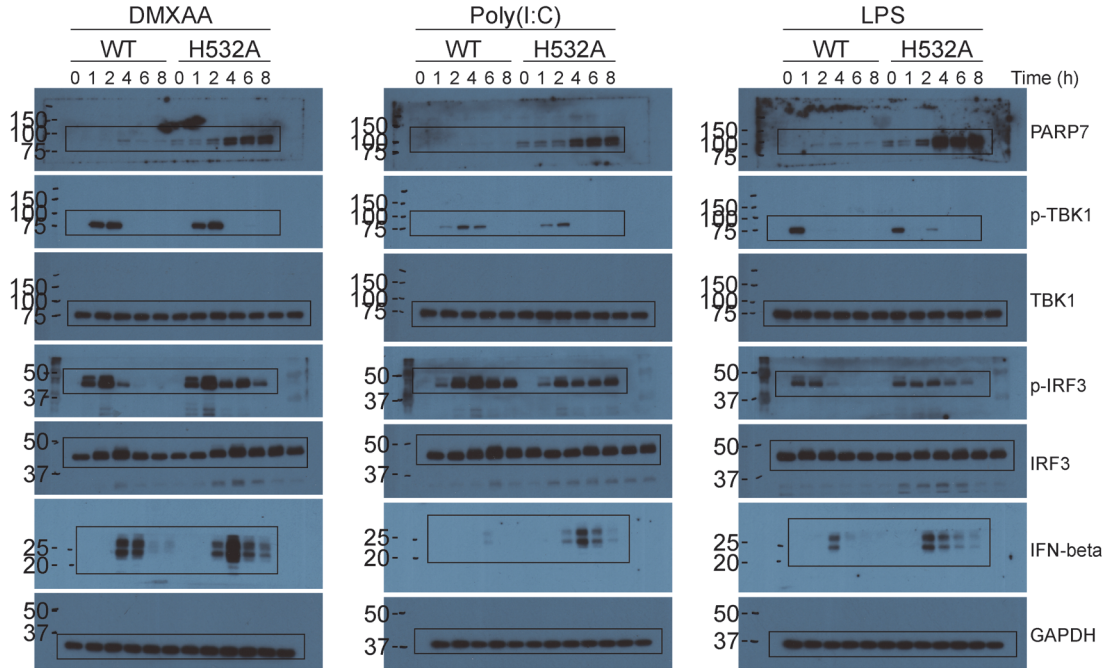

Figure 2J:

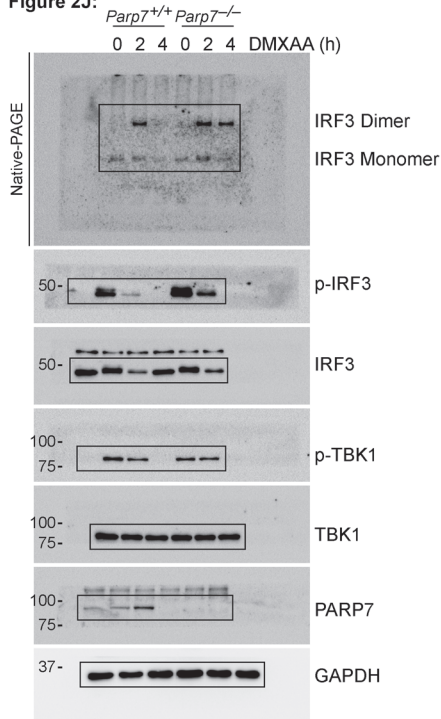

Supplement: SourceData F2 — is the source file for Fig. 2. [file jem_20241184_sourcedataf2.pdf]

SOURCE DATA: FIGURE 3

Figure 3I:

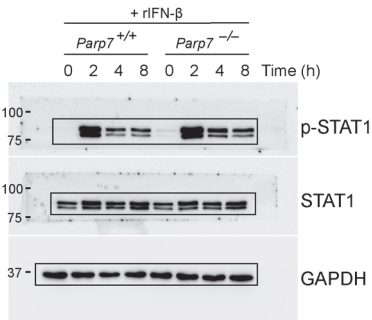

Supplement: SourceData F3 — is the source file for Fig. 3. [file jem_20241184_sourcedataf3.pdf]

SOURCE DATA: FIGURE 5

Figure 5D:

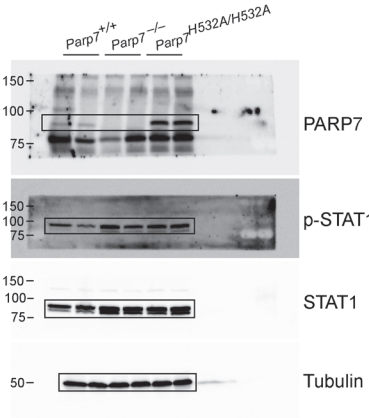

Figure 5I:

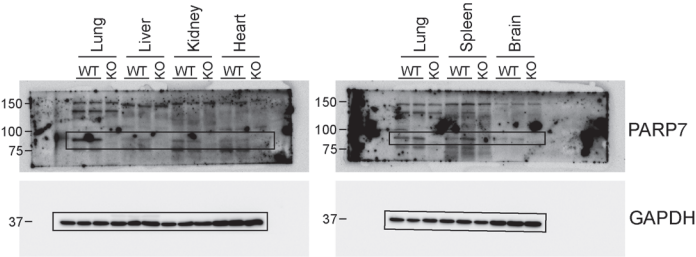

Figure 5J:

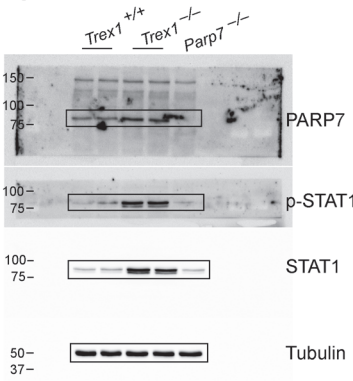

Supplement: SourceData F5 — is the source file for Fig. 5. [file jem_20241184_sourcedataf5.pdf]

SOURCE DATA: FIGURE 6

Figure 6B:

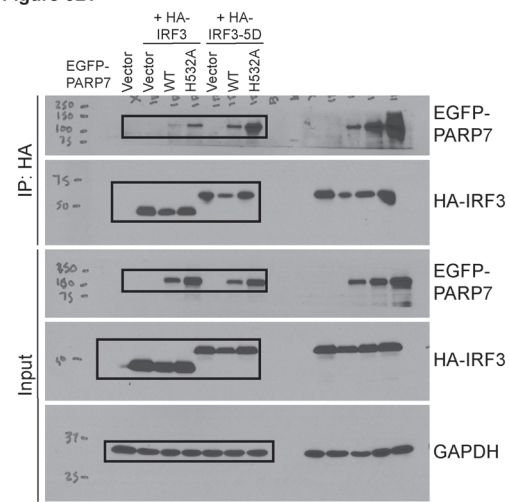

Figure 6C:

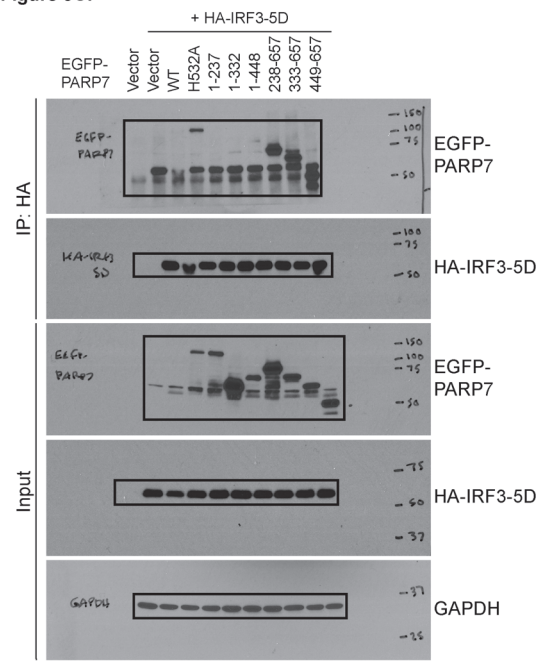

Supplement: SourceData F6 — is the source file for Fig. 6. [file jem_20241184_sourcedataf6.pdf]

SOURCE DATA: FIGURE 7

Figure 7A:

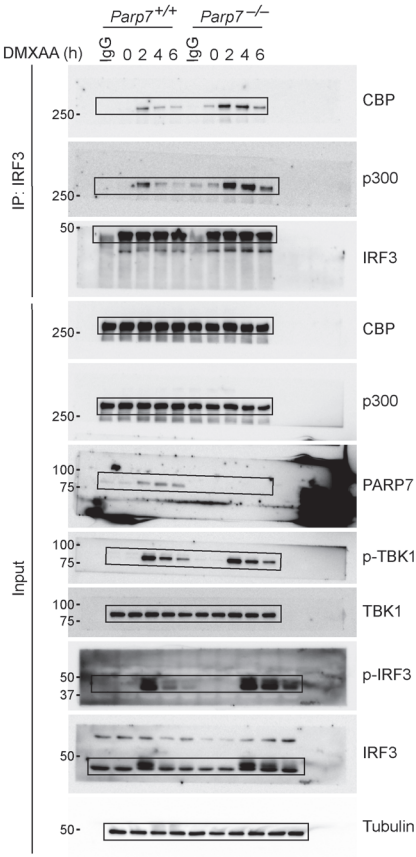

Figure 7B:

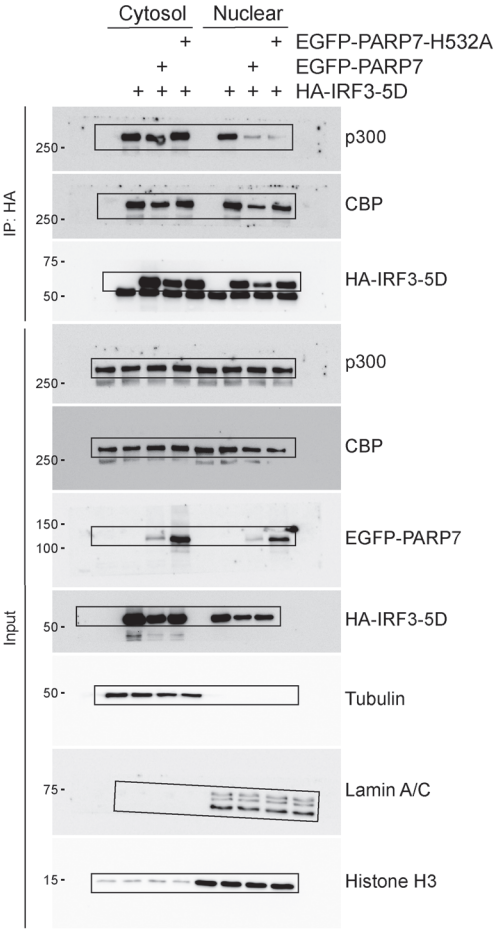

Supplement: SourceData F7 — is the source file for Fig. 7. [file jem_20241184_sourcedataf7.pdf]
