# Supplementary material for: PARP7 inhibits type I interferon signaling to prevent autoimmunity and lung disease
Source: J Exp Med. 2025 Feb 19;222(5):e20241184. doi: 10.1084/jem.20241184 (PMC11837972; doi:10.1084/jem.20241184)

SOURCE DATA: SUPPLEMENTARY FIGURE 4

Supplementary Figure 4H:

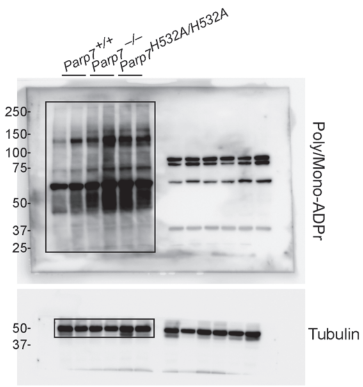

Supplementary Figure 4I:

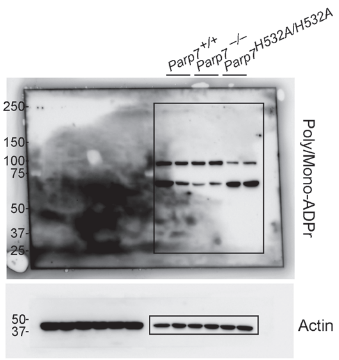

Supplementary Figure 4J:

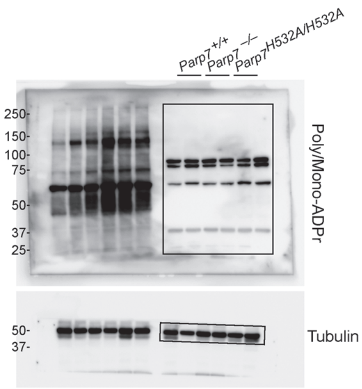

Supplementary Figure 4K:

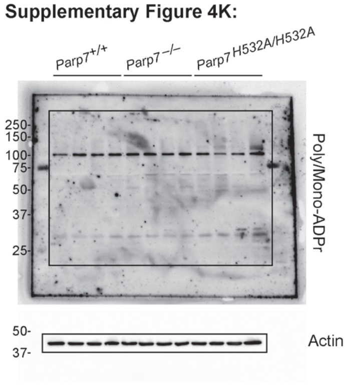

Supplement: SourceData FS4 — is the source file for Fig. S4. [file jem_20241184_sourcedatafs4.pdf]
