# Supplementary material for: PARP7 inhibits type I interferon signaling to prevent autoimmunity and lung disease
Source: J Exp Med. 2025 Feb 19;222(5):e20241184. doi: 10.1084/jem.20241184 (PMC11837972; doi:10.1084/jem.20241184)

SOURCE DATA: SUPPLEMENTARY FIGURE 5

Supplementary Figure 5A:

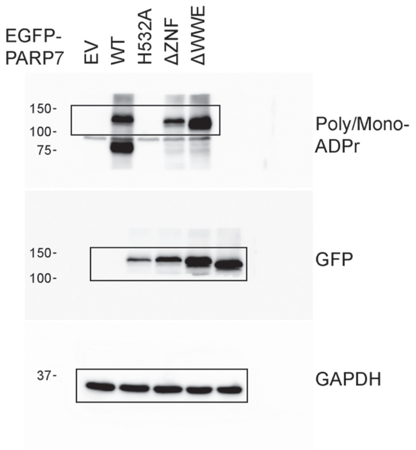

Supplementary Figure 5B:

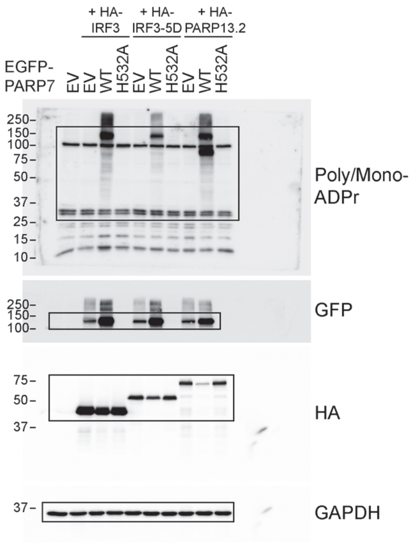

Supplementary Figure 5C:

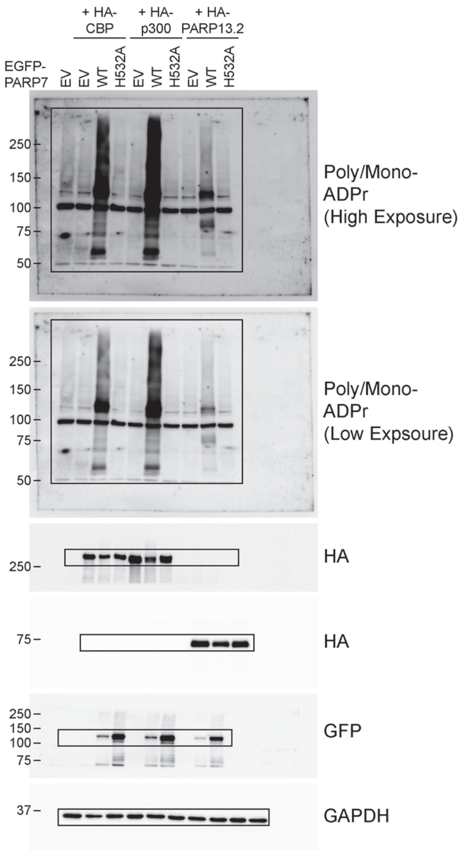

Supplementary Figure 5D:

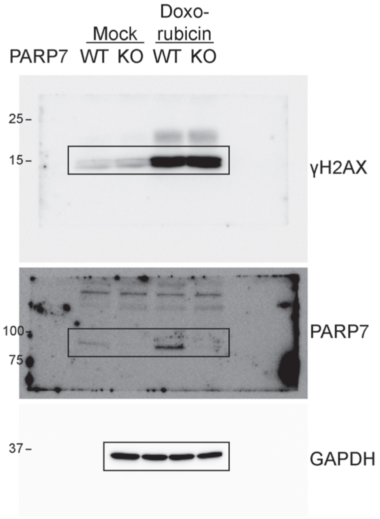

Supplementary Figure 5E:

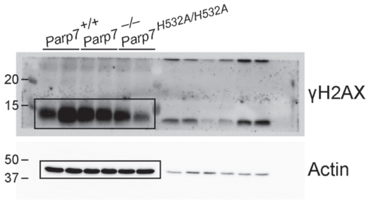

Supplementary Figure 5F:

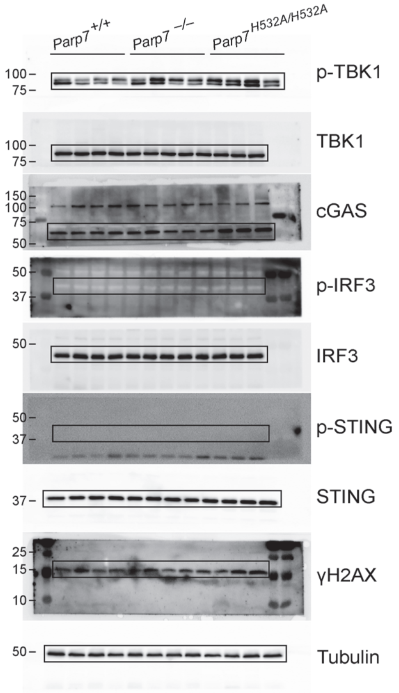

Supplement: SourceData FS5 — is the source file for Fig. S5. [file jem_20241184_sourcedatafs5.pdf]
